# Inter- and Intraindividual Differences in the Capacity of the Human Intestinal Microbiome in Fecal Slurries to Metabolize Fructoselysine and Carboxymethyllysine

**DOI:** 10.1021/acs.jafc.2c05756

**Published:** 2022-09-07

**Authors:** Katja C. W. van Dongen, Clara Belzer, Wouter Bakker, Ivonne M. C. M. Rietjens, Karsten Beekmann

**Affiliations:** † Division of Toxicology, Wageningen University and Research, P.O. Box 8000, Wageningen 6700 EA, The Netherlands; ‡ Laboratory of Microbiology, Wageningen University and Research, P.O. Box 8033, Wageningen 6700 EH, The Netherlands; § Wageningen Food Safety Research (WFSR), Part of Wageningen University and Research, P.O. Box 230, Wageningen 700 AE, The Netherlands

**Keywords:** advanced glycation end product, 16S rRNA analysis, interindividual differences, intraindividual differences, human gut microbiota, new approach methodologies, temporal variability

## Abstract

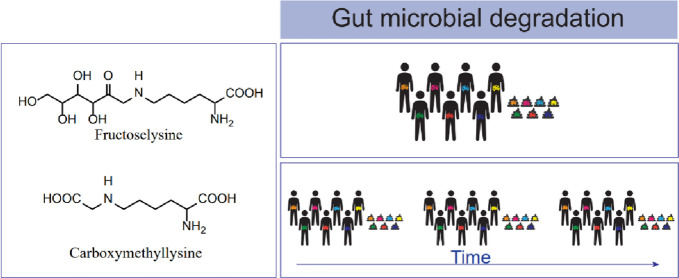

The advanced glycation endproduct carboxymethyllysine and its precursor fructoselysine are present in heated, processed food products and are considered potentially hazardous for human health. Upon dietary exposure, they can be degraded by human colonic gut microbiota, reducing internal exposure. Pronounced interindividual and intraindividual differences in these metabolic degradations were found in anaerobic incubations with human fecal slurries in vitro. The average capacity to degrade fructoselysine was 27.7-fold higher than that for carboxymethyllysine, and degradation capacities for these two compounds were not correlated (*R*^2^ = 0.08). Analysis of the bacterial composition revealed that interindividual differences outweighed intraindividual differences, and multiple genera were correlated with the individuals’ carboxymethyllysine and fructoselysine degradation capacities (e.g., *Akkermansia*, *Alistipes*).

## Introduction

Glycation products are formed during heat processing and storage of food products and are abundantly present in the Western diet^1,2^ and include advanced glycation end products (AGEs) and their precursors such as Amadori products. They are formed by nonenzymatic glycation reactions between amino acids and reducing sugars (i.e., the Maillard reaction^3^) and can be present in food in both their protein-bound as well as in their free forms.^1,2^ One of the most abundant AGEs in the Western diet is carboxymethyllysine, which can be formed via oxidation of the Amadori product fructoselysine^4^ or via reactions of reactive dicarbonyls (i.e., glyoxal and 3-deoxyglucosone) with the amino acid lysine.^5,6^ Dicarbonyls can again be formed via various pathways, including lipid oxidation.^7^ Besides the presence of AGEs in the diet, they are also formed endogenously in the human body.^1,2^ Dietary exposure to AGEs is reported to contribute to AGE levels in plasma^8−11^ and tissues.^11−13^ In addition, exposure to dietary AGEs has been associated with increased markers of negative health effects such as inflammation and endothelial dysfunction.^14^ However, the actual contribution of dietary AGEs toward adverse health effects remains debated.^15^ In addition, alterations in gut bacterial profiles are reported to be induced by exposure to heat-treated diets which are high in AGEs.^11,16−21^ The gut microbiota can also affect AGEs by metabolism thereof. It has been shown that human gut bacteria in fecal slurries—either pooled or individual—can degrade AGEs and their precursors in vitro under anaerobic conditions,^22,23^ with single bacterial strains reportedly able to metabolize specific AGEs such as carboxymethyllysine^24,25^ and its precursor fructoselysine.^26,27^ Because of the potential hazardous effects of AGEs on human health, gut microbial metabolism could serve as a metabolization or bioremediation^28^ pathway potentially leading to detoxification—depending on metabolite formation and the contribution to toxicity of the parent compound—since at least part of the dietary fructoselysine and/or carboxymethyllysine can reach the colon.^29^ Furthermore, it has been shown that substantial interindividual differences in human bacterial degradation of fructoselysine exist,^23^ and also interindividual differences in carboxymethyllysine degradation have been reported in a number of individuals.^22,24^

The human gut microbiota is a complex and dynamic ecosystem with large interindividual differences in composition. The microbiota composition is mainly shaped by environmental factors (e.g., diet and lifestyle),^30^ and it can change over time and, among others, following xenobiotic exposure, which can have consequences for its functioning.^31^ While the most fundamental metabolic functions of the microbiota are considered to be temporally stable and conserved among individuals despite differences in composition,^31^ in a previous study, we reported large interindividual differences in the kinetics of gut microbial degradation of fructoselysine.^23^ Given the dynamic nature of the microbiota^32^ and being reactive to a plethora of host and environmental factors, it is of interest to also assess the temporal variability of gut microbial degradation activities. Therefore, in the present study, we aim to quantify interindividual differences as well as intraindividual differences of gut microbial degradation of fructoselysine and carboxymethyllysine and compare their metabolism. To this end, in vitro anaerobic incubations with fecal samples collected from multiple individuals at different sampling times spread over 3 to 16 weeks were performed with both fructoselysine and carboxymethyllysine as substrates which is a relevant and novel addition to previous research. In addition, total bacterial cell load in these fecal samples was quantified and microbial composition was characterized by 16S rRNA amplicon sequencing and correlated with the respective degradation capacities.

## Materials and Methods

### Chemicals and Reagents

Carboxymethyllysine (CAS: 5746-04-3) and *N*-ε-fructoselysine (fructoselysine; CAS: 21291-40-7) were purchased from Carbosynth Limited (Berkshire, UK). D4-labelled carboxymethyllysine was purchased from Buchem BV (Apeldoorn, the Netherlands). Glycerol (CAS: 56-81-5) was purchased from Sigma-Aldrich (Steinheim, Germany). Formic acid (99–100%, analytical grade, CAS: 64-18-6) was purchased from Merck (Darmstadt, Germany). Phosphate-buffered saline (PBS) was purchased from Gibco (Paisley, UK). Acetonitrile (ACN; UPLC/MS grade; CAS: 75-05-8) was obtained from BioSolve BV (Valkenswaard, the Netherlands).

### Collection of Human Fecal Samples

Fresh fecal samples were collected from 20 human volunteers (11 females, 9 males), aged between 19 and 64 years at sampling time one (ST1). Of these individuals, 13 donated a sample again on two later occasions (8 females, 5 males, aged between 24 and 65 years old, with ≥3 weeks in-between over a total maximum period for all 3 donations of 16 weeks, corresponding to ST2 and ST3; see Supporting Information Table S1 for the sampling times of each individual). Volunteers were not pregnant, did not suffer from chronic gastrointestinal diseases, and did not use antibiotics 3 months prior to donation. Fecal samples were immediately processed after donation as described before^23^ and stored at −80 °C after a 4× dilution (w/v) in anaerobic storage buffer consisting of 10% glycerol in PBS until further use. All participants granted informed consent before participation in this study. The study design was assessed by the Medical Ethical Committee of Wageningen University and judged to not fall under the Dutch “Medical Research Involving Human Subjects Act.”

### Anaerobic Incubations of Fecal Slurries with Carboxymethyllysine and Fructoselysine

Anaerobic incubations with human fecal slurries and fructoselysine or carboxymethyllysine were performed as previously described.^23^ In short, pooled or individual human fecal slurries were mixed with anaerobic PBS and carboxymethyllysine or fructoselysine in optimized experimental conditions as described in more detail below. 50 μL of this mixture was divided over Eppendorf tubes and incubated at 37 °C for the required duration, after which reactions were stopped by addition of 50 μL ice-cold ACN and stored on ice for >15 min. All handlings were performed inside an anerobic chamber (85% N_2_, 10% CO_2_ and 5% H_2_) (Bactron EZ anaerobic chamber). Incubations were performed in anaerobic PBS to avoid bacterial growth and keep the bacterial composition as close to the original fecal composition as possible. Given that this use of PBS limits the available nutrients to those present in the fecal samples, incubations were carried out for only a limited time period namely up to at most 3 h. Samples were centrifuged at 15,000 × *g* for 15 min at 4 °C, and the resulting supernatants were used for subsequent analysis by liquid chromatography–tandem mass spectrometry (LC–MS/MS).

The experimental conditions that have been applied for the anaerobic incubations with individual human fecal slurries were optimized using pooled human fecal slurries. This was done to achieve linear reactions over the amount of fecal slurry and over time, with incubation times as short as possible to remain as close as possible to the human fecal material. Saturating substrate concentrations were selected to facilitate detection of maximum degradation rates and comparison between all performed anaerobic incubations using individual human fecal slurries. For fructoselysine, the optimization of experimental conditions was previously described,^23^ resulting in anaerobic incubations of 1 h with 5% individual fecal slurry in PBS (i.e., 0.0125 g/mL) with a final, saturating substrate concentration of 125 μM fructoselysine (being ≥2-fold higher than the *K*_m_ of the pooled human fecal slurries). For carboxymethyllysine degradation, experimental conditions were optimized with pooled human fecal samples containing equal amounts of 20 individual human fecal samples collected at ST1. With this pooled fecal slurry, experimental conditions were optimized to achieve linear degradation of carboxymethyllysine over increasing percentage of fecal slurry and over time (Supporting Information Figure S1A). Saturating substrate concentrations were selected (Supporting Information Figure S1B). This resulted in the anaerobic incubations of individual fecal samples being performed with 20% individual human fecal slurries in PBS (i.e., 0.05 g/mL) for 3 h with a final, saturated substrate concentration of 80 μM carboxymethyllysine. All experiments were performed in at least technical duplicates and were repeated three times. Control incubations of the substrate in PBS showed that the substrates were stable without addition of human fecal slurries (Supporting Information Figure S1C). Blank incubations (anaerobic incubations without the substrate) showed that no background release of fructoselysine or carboxymethyllysine occurred during the anaerobic incubations with human fecal slurry (data not shown).

### Quantification of Fructoselysine and Carboxymethyllysine by LC–MS/MS

80 μL of supernatants of the anaerobic fecal slurry incubations were transferred into LC–MS/MS vials. In addition, for carboxymethyllysine, 10 μL of 120 μM aqueous D4-carboxymethyllysine was added as the internal standard. Fructoselysine and carboxymethyllysine concentrations were quantified using a Shimadzu Nexera XR LC-20AD SR UPLC system coupled to a Shimadzu LCMS-8040 triple quadrupole mass spectrometer (Kyoto, Japan). The LCMS-8040 coupled with an ESI source was used for MS/MS identification. Positive ionization for multiple reaction monitoring (MRM) mode was used. For fructoselysine, 2 μL of supernatant was injected onto a Phenomenex Polar-RP Synergi column (30 × 2 mm, 2.5 μm), at which fructoselysine eluted at 5.6 min and was quantified using the precursor to product transition *m/z* 309.2 > 84.2 [collision energy (CE) = −31 V], which was the most intense fragment ion, as previously described.^23^ For carboxymethyllysine, 1 μL of supernatant was injected onto a Waters Acquity BEH Amide column (2.1 × 100 mm, 1.7 μm) at 40 °C. The mobile phase consisted of a gradient made from solvent A [i.e., ultrapure water with 0.1% formic acid (v/v)] and solvent B [i.e., ACN with 0.1% formic acid (v/v)]. The gradient started with 75% B, reached 12.5% B at 5 min, and was subsequently kept at 12.5% B until 11 min, followed by a shift to reach 95% B at 12 min, which was kept stable until 17 min before returning to the initial start conditions at 18 min keeping these conditions up to 24 min. The initial flow of 0.3 mL/min was decreased to 0.15 mL/min from 5 to 5.5 min and remained 0.15 mL/min up to 11 min before returning to the initial flow of 0.3 mL/min at 17 min. Under these conditions, carboxymethyllysine and D4-carboxymethyllysine eluted at 4.6 min. Carboxymethyllysine was quantified using the precursor to product transition *m/z* 204.9 > 84.2 (CE = −21 V). MRM transitions *m/z* 204.9 > 130.2 (CE = −12 V) and *m/z* 204.9 > 56.1 (CE = −39 V) were used as reference ions. D4-Carboxymethyllysine was quantified using the MRM transition *m/z* 208.9 > 88.1 (CE = −21 V), while MRM transitions *m/z* 208.9 > 134.2 (CE = −12 V) and *m/z* 208.9 > 56.1 (CE = −42 V) were used as reference ions. For quantification of carboxymethyllysine concentrations present in the samples, the area ratio of carboxymethyllysine and a known concentration of D4-carboxymethyllysine was determined and further quantified via the area ratios of an external calibration curve which was prepared in the same way as the incubation samples using commercially available carboxymethyllysine and D4-carboxymethyllysine. Concentrations of fructoselysine present in the samples were achieved by an external calibration curve for which samples were prepared in the same way as the fecal incubation samples using commercially available fructoselysine. For fructoselysine, use of an internal standard was not essential because the matrix effect of the fecal material was shown to be negligible when comparing fructoselysine calibration curves made in PBS or in fecal slurries. For carboxymethyllysine, a matrix effect was more obvious due to the higher concentration of fecal slurry applied in these incubations. Peak areas were integrated using LabSolutions software (Shimadzu). The amount of degraded fructoselysine or carboxymethyllysine during incubation was calculated and expressed in μmol degraded/g feces/hour and μmol degraded/1 × 1012 bacterial cells/h.

### Bacterial Taxonomic Profiling by 16S rRNA Gene Amplicon Sequencing

DNA was isolated from the fecal slurries using a bead-beating procedure in combination with the customized MaxWell 16 Tissue LEV Total RNA Purification Kit (XAS1220; Promega Biotech AB, Stockholm, Sweden). DNA isolates underwent triplicate polymerase chain reaction (PCR) reactions of the 16S ribosomal RNA (rRNA) gene V4 region (515-F; 806-R) with a library approach as described before.^11,23^ PCR products were purified, pooled, and sequenced (Illumina NovaSeq 6000, paired-end, 70 bp; Eurofins Genomics Europe Sequencing GmbH, Konstanz, Germany).

### Total Bacterial Load by Quantitative PCR

To quantify the total bacterial load in each individual fecal slurry, quantitative PCR (qPCR) was performed based on a previously described method.^33^ Triplicate qPCR reactions consisted of 1 μL of DNA isolate (1 ng/μL) and 9 μL of reaction mixture (composed of 62.5% iQ SYBR Green Supermix), 2.5% forward primer (10 μM), 2.5% reverse primer (10 μM), and 32.5% nuclease-free water. The following set of primers for total bacterial 16S rRNA genes were used: 1369-F (5′-CGG TGA ATA CGT TCY CGG-3′) and 1492-R (5′-GGW TAC CTT GTT ACG ACT T-3′). A purified DNA isolate of *Escherichia coli* was used to create a standard curve to facilitate quantification. The amplification program started at 95 °C for 10 min, followed by 40 cycles of denaturing at 95 °C for 15 s, annealing at 60 °C for 30 s, and elongation at 72 °C for 15 s. The program ended with a melt curve from 60 °C to 95 °C. A CFX-384 Touch Real-Time PCR detection system (Bio-Rad, California, USA) was used. Data analysis was performed with the CFX manager (Bio-Rad). Quantified copy numbers of total 16S rRNA genes/g fecal sample were divided by the average 16S rRNA genes per bacterium (i.e., 4.2^34^) and thus transformed to total bacterial load/g fecal sample.

### Data Analysis

Sequences of the 16S rRNA gene were analyzed using NG-Tax 2.0 pipeline with default settings,^35^ generating *de novo* exact match sequence clusters (ASVs; amplicon sequence variants). The SILVA 16S rRNA gene reference database^36^ release 132 was used to assign taxonomy. R (version 4.0.2) was used for further data analysis using the Phyloseq package^37^ (version 1.34.0) to combine the ASV table with the phylogenetic tree and metadata. A relative abundance cutoff of 0.1% of a taxa in one of the individual samples was used to include ASVs for further analyses, unless mentioned otherwise. When desired, relative abundance data were transformed into absolute abundance data by multiplying the relative abundance of a taxa within one sample with the corresponding total bacterial load/g fecal sample, as quantified by qPCR. Microbiome package^38^ (version 1.12.0) was used to create composition plots of the top taxa present in the samples sorted with hierarchical clustering based on Bray–Curtis beta diversity dissimilarities using the average linkage approach using all taxa present in the samples. Belonging dendrograms were created with the packages Phyloseq,^37^ Stats, and Ape^39^ (version 5.4.1). Spearman’s rank correlations of fructoselysine and carboxymethyllysine degradation with microbial taxa which were present at a relative abundance of >1% in one of the samples and glomerated at genus level were made. *P* values were adjusted for multiple testing with the Benjamini & Hochberg false discovery rate (FDR) using the Microbiome package.^38^

Quantified amounts of degraded fructoselysine and carboxymethyllysine of three repeated experiments were averaged, and standard deviations were calculated using GraphPad Prism 5.0. Individual amounts of degraded fructoselysine and carboxymethyllysine were corrected for the applied weight of feces used in the incubations and/or the total bacterial cell load per gram feces and expressed per hour. For the latter, the percentage of total substrate degraded was quantified using the total substrate added relative to the average total bacterial cell load set as 100%. Outliers were identified using IBM SPSS Statistics version 25 using a multiplier of 3.0. Statistically significant differences in the amount of fructoselysine or carboxymethyllysine degraded per sampling time were assessed with an ANOVA test combined with a Tukey’s multiple comparison post hoc test. Unless otherwise stated, results were found to be statistically significant when *P*-values were <0.05.

## Results

### Interindividual and Intraindividual Differences in Gut Microbial Carboxymethyllysine Degradation Profiles In Vitro

**Figure 1:**
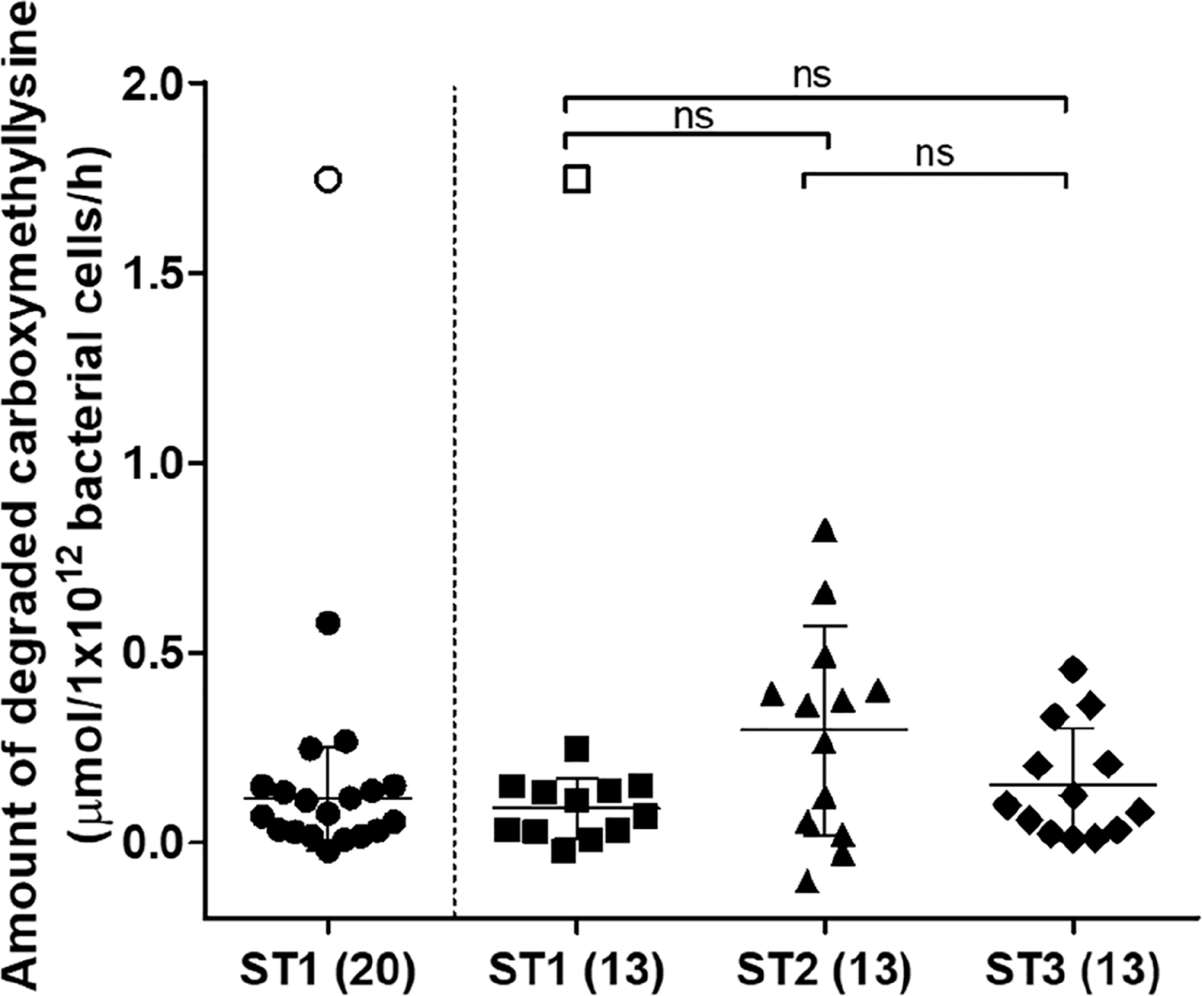
Amount of carboxymethyllysine degraded after anaerobic incubation of individual human fecal samples (0.05 g/mL final concentration) with 80 μM carboxymethyllysine, expressed per hour. ST1, ST2, and ST3 indicate different sampling times, and the number in brackets refers to the number of individuals who donated at these different sampling times. For ST1, this is a total of 20 individuals, whereof 13 individuals donated at two additional sampling times, which are separately visualized in the second column ST1(13). Scatter dots indicate average values of three independent experiments for each individual fecal sample. Center bars and whiskers indicate mean values with the standard deviation. Open symbols refer to an identified outlier. N.s. refers to not statistically significant.

Interindividual differences in carboxymethyllysine degradation were investigated for all collected individual fecal samples, that is, 20 individual fecal samples donated at a first sampling time (i.e., ST1) and fecal samples donated by 13 of these 20 individuals at two other sampling times (i.e., ST2 and ST3). Anaerobic incubations were performed with individual human fecal slurries based on optimized experimental conditions (i.e., with final concentrations of 0.05 g feces/mL and 80 μM carboxymethyllysine; see Supporting Information Figure S1). The amount of carboxymethyllysine degraded per hour was expressed relative to the total bacterial load in the samples as quantified by qPCR, which were in line with the literature^40^ (for bacterial load of samples, see Supporting Information Figure S2; for carboxymethyllysine degradation per g feces, see Supporting Information Figure S3). For individual 1, ST1 was assessed as being an outlier and thus excluded from further analyses. Overall, the degradation capacities of the individual fecal slurries tested ranged from no or minimal carboxymethyllysine degradation to a maximum of 0.83 μmol carboxymethyllysine degradation/1 × 10^12^ bacterial cells/h (Individual 5, ST2), the latter resulting in 65% of the added substrate being degraded under the experimental conditions applied. Average carboxymethyllysine degradation for the different sampling times for the 13 individuals that donated three times were not significantly different and were in the same range, that is, 0.09 (ST1), 0.3 (ST2), and 0.15 (ST3) μmol carboxymethyllysine degradation/1 × 10^12^ bacterial cells/h (Figure 1), resulting in 7, 24, and 12% of the added substrate being degraded under the experimental conditions applied, respectively. Background levels of carboxymethyllysine in the individual fecal slurries ranged from 0.5–5 μM and were not correlated with the amount of degraded carboxymethyllsyine (*R*^2^ = 0.075, see Supporting Information Figure S4A).

**Figure 2:**
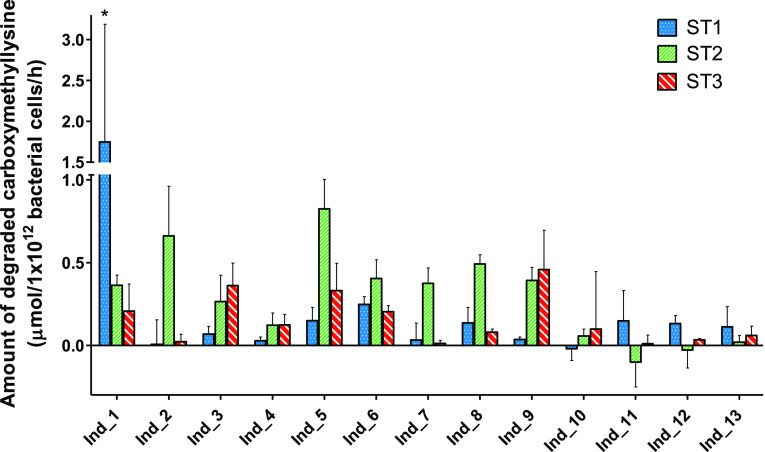
Intraindividual differences in the degradation of carboxymethyllysine upon anaerobic incubations with individual human fecal slurries (final concentration of 0.05 g/mL), shown for 13 individual donors sampled at three sampling times (i.e., ST1, ST2 and ST3). The data represent the average ± SD of three repeated experiments. * Refers to Ind 1 ST1 which was assessed as an outlier.

Intraindividual variability in carboxymethyllysine degradation capacities was further quantified for the 13 individuals who donated at ST1, ST2, and ST3, as shown in Figure 2. The capacity to degrade carboxymethyllysine differed within most individuals over time. As such, several individuals were not always able to degrade carboxymethyllysine (i.e., individuals 2, 4, 7, 10, 11, and 12), while other individuals mainly showed differences in the amount being degraded (i.e., individuals 1, 3, 5, 6, 8, 9, and 13). The largest absolute difference of carboxymethyllysine being degraded was for individual 5 with a difference of 0.68 μmol/1 × 10^12^ bacterial cells/h between sampling time ST1 and ST2, corresponding to a difference of 54% of the added substrate being degraded under the experimental conditions applied. Almost no difference of carboxymethyllysine degradation between different sampling times was detected for individual 4 between sampling times ST2 and ST3 (with a negligible difference of 0.002 μmol/1 × 10^12^ bacterial cells/h).

When expressing the degradation per gram feces instead of bacterial load (see Supporting Information Figure S3), inter- and intraindividual differences ranged from no to minimal carboxymethyllysine degradation to a maximum of 0.48 μmol carboxymethyllysine/g feces/h being degraded, the latter resulting in 91% of the added substrate being degraded under the experimental conditions applied. Comparing the amount of bacterial cells/g feces with the amount of degraded carboxymethyllysine/g feces/h confirms that there is no correlation between the absolute number of bacteria in the samples and the ability to degrade carboxymethyllysine (*R*^2^ = 0.059; see Supporting Information Figure S5A).

### Interindividual Differences and Intraindividual Differences in Fructoselysine Human Gut Microbial Degradation Profiles In Vitro

**Figure 3:**
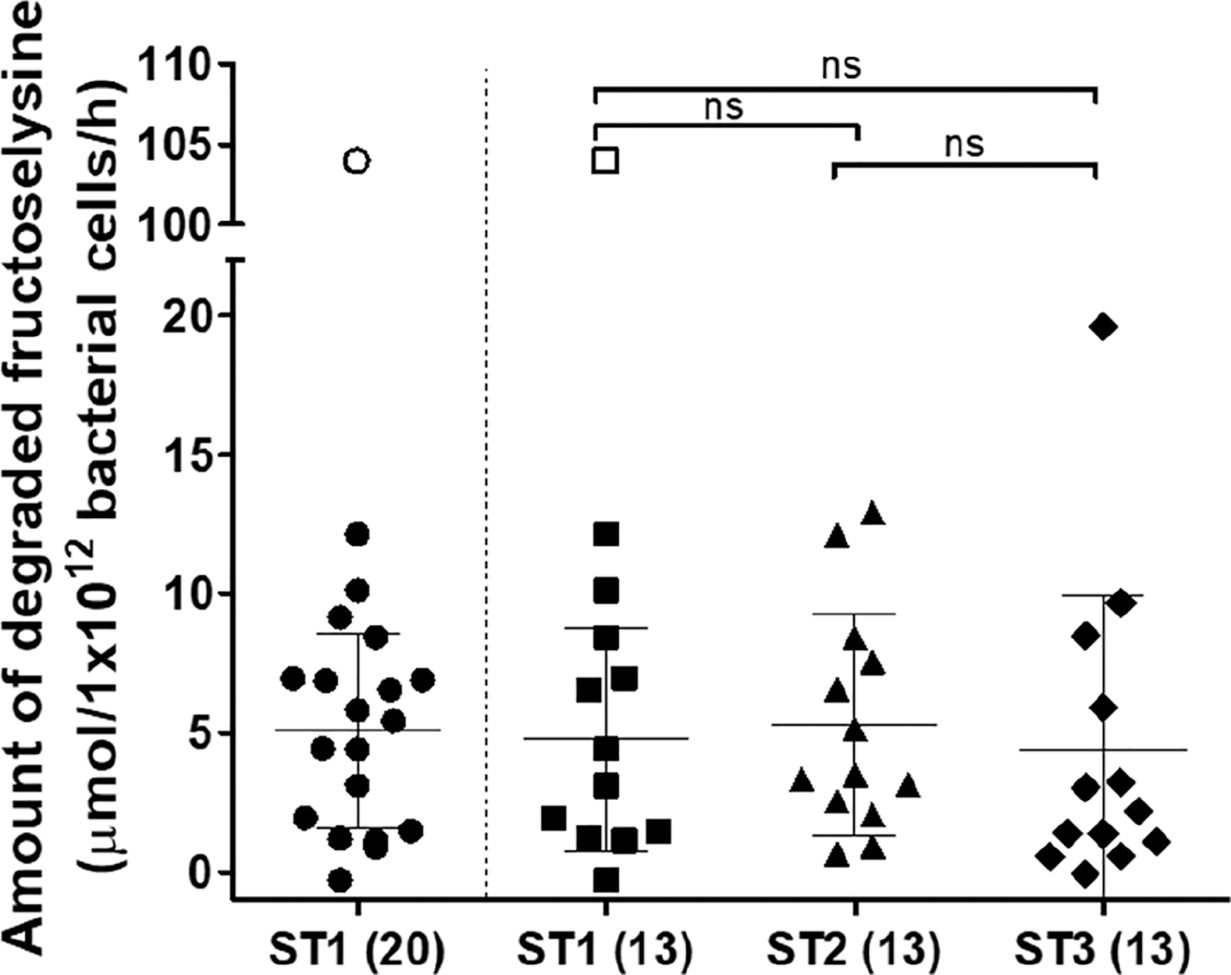
Amount of fructoselysine degraded after anaerobic incubation of individual human fecal samples (0.0125 g/mL final concentration) with 125 μM fructoselysine per hour. ST1, ST2, and ST3 indicate different sampling times, and the number in brackets refers to the number of individuals who donated at these different sampling times. For ST1, this is a total of 20 individuals, whereof 13 individuals donated at two additional sampling times, which are separately visualized in the second column ST1(13). Scatter dots indicate average values of three independent experiments. Center bars and whiskers indicate mean values with the standard deviation. Open symbols refer to an identified outlier. N.s. refers to not statistically significant.

To allow comparison of carboxymethyllysine degradation to degradation of its precursor fructoselysine, interindividual and intraindividual differences in fructoselysine degradation were quantified using previously optimized experimental conditions^23^ at a saturated substrate concentration of fructoselysine (i.e., with final concentrations of 0.0125 g feces/mL and 125 μM of fructoselysine) using the same individual human fecal samples as for carboxymethyllysine. The amount of fructoselysine degraded per hour was expressed relative to the total bacterial load in the samples as quantified by qPCR (for bacterial load of samples, see Supporting Information Figure S2; for fructoselysine degradation per g feces, see Supporting Information Figure S6). The value from Individual 1, ST1, was assessed as an outlier and therefore excluded from further analyses. Overall, interindividual differences in the degradation capacity of the individual fecal slurries tested ranged from no or only minimal degradation to 19.63 μmol fructoselysine degradation/1 × 10^12^ bacterial cells/h (individual 10, ST3), the latter resulting in 82.5% of the added substrate being degraded under the experimental conditions applied. Average fructoselysine degradation for the different sampling times for the 13 individuals that donated three times was not statistically significantly different and was in the same range, that is, 4.8 (ST1), 5.3 (ST2), and 4.4 (ST3) μmol/1 × 10^12^ bacterial cells/h (Figure 3), resulting in 20, 22, and 18% of the added substrate being degraded under the experimental conditions applied, respectively. Background levels of fructoselysine in the individual fecal slurries ranged from 4.7–11.5 μM and were not correlated with the amount of degraded fructoselysine (*R*^2^ = 0.001; see Supporting Information Figure S4B).

**Figure 4:**
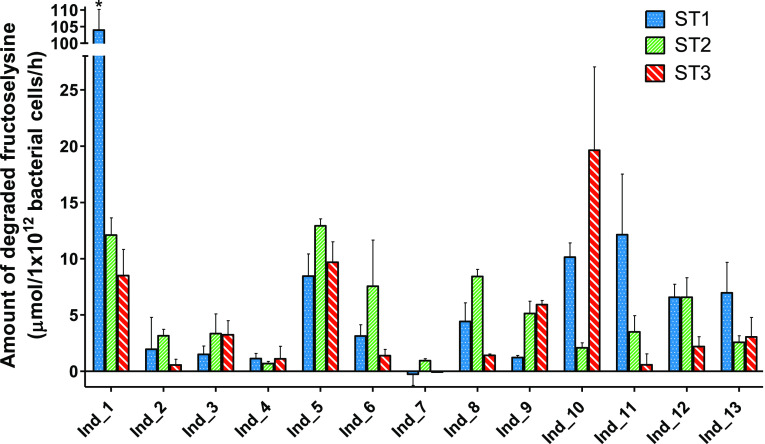
Intraindividual differences in the degradation of fructoselysine upon anaerobic incubations with individual human fecal slurries (final concentration of 0.0125 g/mL), shown for 13 individual donors sampled at three sampling times (i.e., ST1, ST2 and ST3). The data represent the average ± SD of three repeated experiments * Refers to Ind 1 ST1 which was identified to be an outlier.

Intraindividual variability in fructoselysine degradation capacities was further quantified within the 13 individuals who donated at ST1, ST2, and ST3, as shown in Figure 4. The capacity to degrade fructoselysine differed within most individuals over time; all samples from individual 7 degraded no or only very little fructoselysine, and two individuals showed a relatively stable capacity to degrade fructoselysine for all three sampling times (i.e., individuals 4 and 5). The largest absolute difference of fructoselysine being degraded was within individual 10 with a difference of 17.5 μmol/1 × 10^12^ bacterial cells/h between sampling times ST2 and ST3, corresponding to a difference of 74% of the added substrate being degraded under the experimental conditions applied. Almost no absolute difference in degraded fructoselysine between different sampling times was detected for individual 12 between sampling times ST1 and ST2 (with a difference of 0.01 μmol/1 × 10^12^ bacterial cells/h).

When expressing fructoselysine degradation per gram feces instead of bacterial load (see Supporting Information Figure S6), inter- and intraindividual differences ranged from no to minimal fructoselysine degradation to a maximum of 5.4 μmol fructoselysine/g feces/h being degraded, resulting in 54% of the added substrate being degraded under the experimental conditions applied. Comparing the amount of bacterial cells/g feces with the amount of degraded fructoselysine/g feces/h confirms that there is no correlation between the absolute number of bacteria in the samples and the ability to degrade fructoselysine (*R*^2^ = 0.002; see Supporting Information Figure S5B).

### Comparison of Carboxymethyllysine and Fructoselysine Degradation

Linear regression analysis of the amount of fructoselysine and carboxymethyllysine degraded/1 × 10^12^ bacterial cells/h revealed that there is at best a limited correlation between the capability to microbially degrade fructoselysine and carboxymethyllysine for all individual collected fecal samples (*R*^2^ = 0.084 for degradation/1 × 10^12^ bacterial cells/h, see Figure S7A; for degradation/g feces/h *R*^2^ = 0.253, see Supporting Information Figure S7B). Overall, fructoselysine was degraded faster than carboxymethyllysine (i.e., on average 27.7-fold faster when expressed relative to the bacterial load; 23.4-fold faster when expressed per gram feces). Regarding intraindividual differences, fructoselysine and carboxymethyllysine showed comparable relative variability among all sampled individuals [coefficient of variation(CV) = 85% for fructoselysine; CV = 112% for carboxymethyllysine; *n* = 45].

Assuming a total transit time in the colon of 24 h^41^ and a total fecal mass of 128 g per 24 h,^42^ the experimentally obtained in vitro average degradation capacities of all individuals can be extrapolated to the in vivo situation. This analysis can reveal whether the estimated daily intake (EDI) of carboxymethyllysine and fructoselysine, amounting to 0.3–1.1 mg/kg bw/day for carboxymethyllysine and 7.1–14.3 mg/kg bw/day for fructoselysine,^22,43^ can be completely degraded in the colon (see Supporting Information Table S2).^53^ For carboxymethyllysine, depending on the level of intake, 18–39 of the 46 tested fecal samples had degradation capacities too low to completely degrade the EDI, and 11–19 of the 20 donors who donated a fecal sample (once or more) had degradation capacities too low to completely degrade the EDI at one or more sampling times. For fructoselysine, depending on the level of intake, 8–20 of the 46 tested fecal samples had degradation capacities too low to completely degrade the EDI. 4–10 of the 20 donors who donated a fecal sample (once or more) had degradation capacities too low to completely degrade the EDI at one or more sampling times (see Supporting Information Table S2).

### Interindividual and Intraindividual Differences in Human Gut Microbial Composition

**Figure 5:**
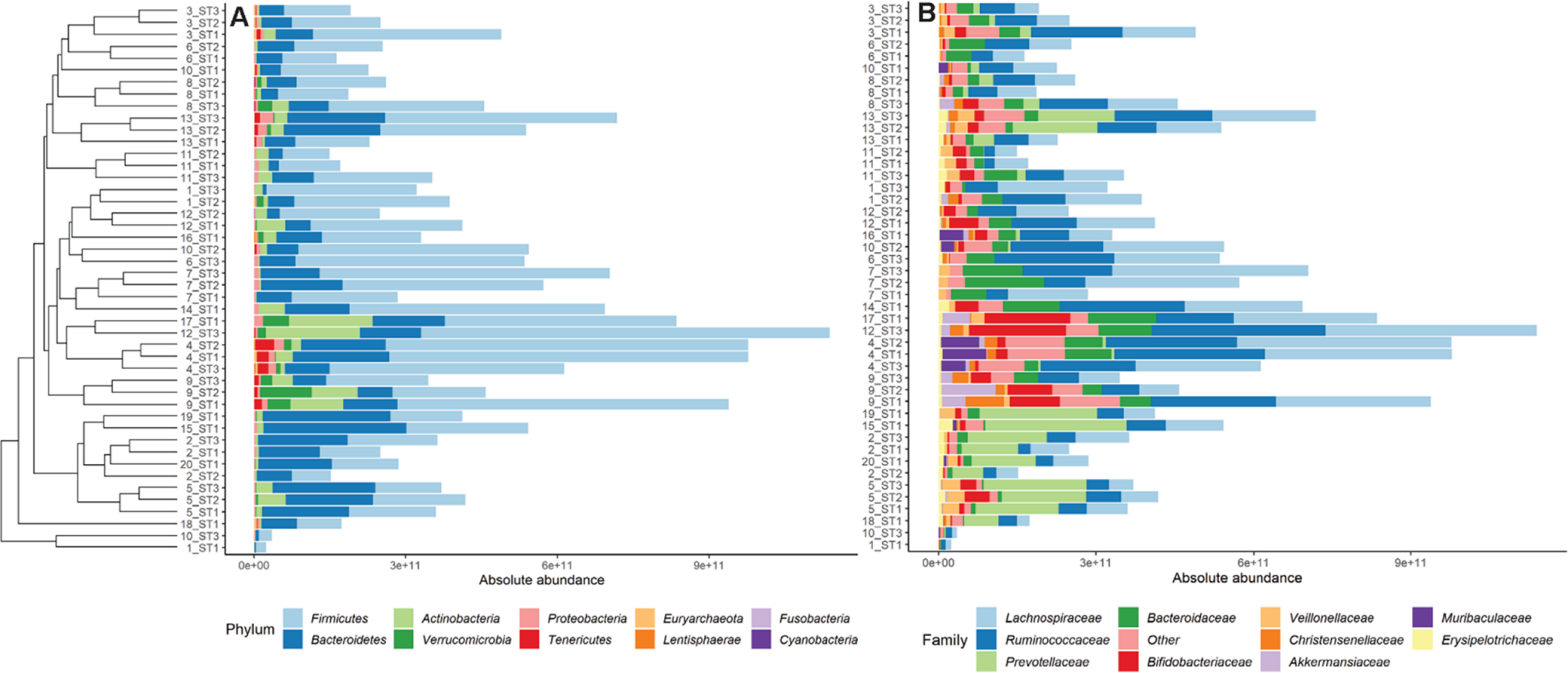
Absolute abundance of microbial taxa, assessed with 16S rRNA amplicon sequencing and qPCR, present in the individual fecal samples (y-axis labels consist of subject number and sampling time). The top 10 taxa present at phylum (panel A) and family (panel B) levels are provided, sorted based on hierarchical clustering of Bray–Curtis dissimilarities using the average linkage approach with all taxa included.

Bacterial taxonomic profiling by 16S rRNA amplicon sequencing revealed interindividual and intraindividual differences in bacterial composition of the collected fecal samples. Bray–Curtis beta diversity dissimilarities (Supporting Information Figure S8) show a variance of 19.7% on the first PCoA axis and a variance of 12.9% on the second PCoA axis. This is in line with the literature,^44^ indicating that the variation observed in the cohort of this study is representative. Composition plots of the absolute abundance of the main taxa at the phylum and family levels (Figure 5) and the genus level (Supporting Information Figure S9) combined with hierarchical clustering of Bray–Curtis beta diversity dissimilarities of the full dataset indicate, with some exceptions, that most individuals clustered together over their three sampling times. This indicates that interindividual differences in the overall microbial composition of the collected samples seem to be larger than intraindividual differences, as reported in the literature.^40,45,46^ Firmicutes appeared to be the highest abundant phylum present in most samples followed by Bacteroidetes. The families *Lachnospiraceae*, *Ruminococcaceae* and for some individuals *Prevotellaceae*, *Bacteroidaceae,* or *Bifidobacteriaceae* accounted for the largest abundance of the microbial taxa present in the collected samples. Supporting Information Figure S10 shows relative abundance data at phylum, family, and genus levels.

### Associations of Bacterial Taxa with Carboxymethyllysine and Fructoselysine Degradation Profiles

**Figure 6:**
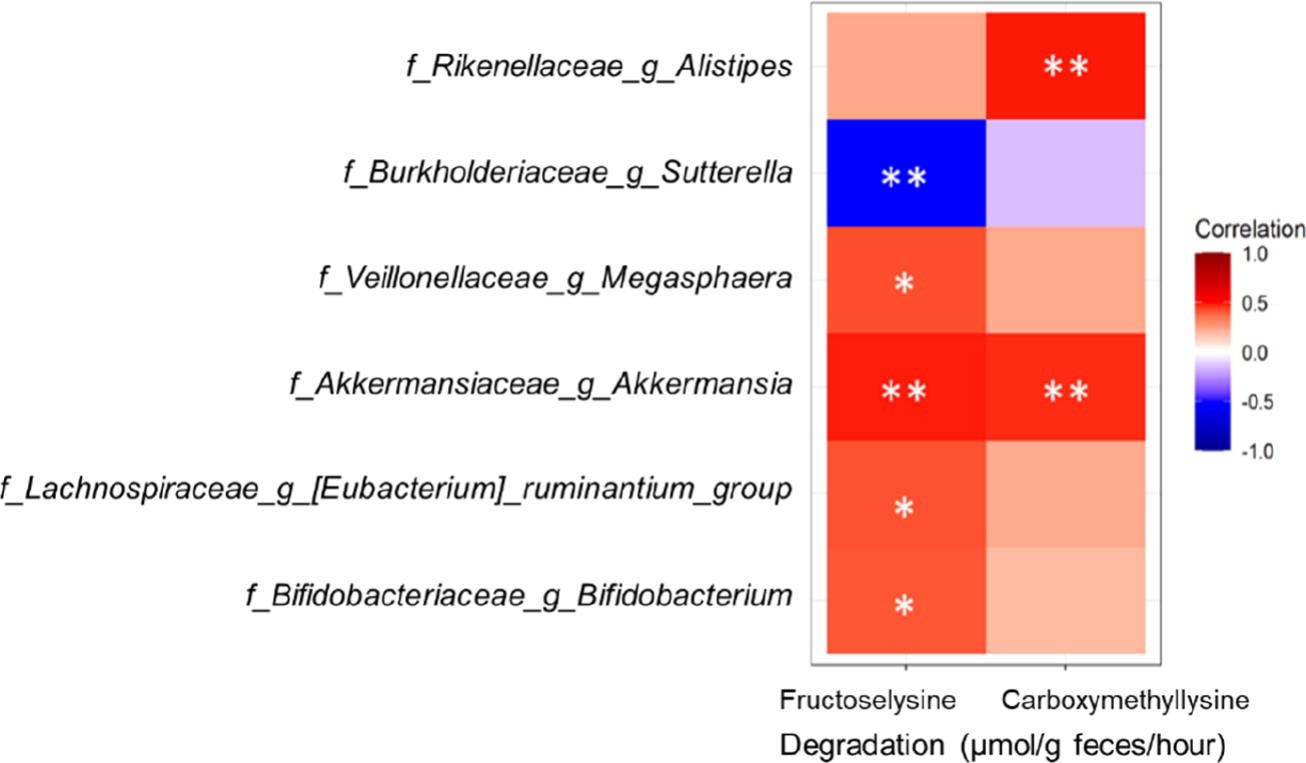
Spearman’s rank correlation analysis of bacterial genera with the amount of degraded fructoselysine and carboxymethyllysine per gram feces per hour. Bacterial genera present with a relative abundance >1% in one of the individual fecal samples were included and were transformed into absolute abundance (using quantified total bacterial cell load by qPCR). Only taxa with one or more statistically significant correlation after correction for multiple testing (FDR) were included in this heatmap and are indicated as follows: ** *P*-value < 0.05; * *P*-value <0.1.

To explore potential relationships between specific bacterial genera and carboxymethyllysine or fructoselysine degradation, a Spearman’s rank correlation analysis was performed with genera present with a relative abundance >1% in one of the individual fecal samples. Based on absolute bacterial abundances as quantified via total bacterial cell load, multiple genera showed a statistically significant correlation with carboxymethyllysine and/or fructoselysine degradation expressed per gram feces/h (Figure 6). The following genera showed a positive correlation with fructoselysine degradation, ordered by increasing the adjusted *P-*value: *Akkermansia* (ρ = 0.49; *P-*value = 0.029), *Megasphaera* (ρ = 0.43; *P-*value = 0.085), *Eubacterium_ruminantium_group* (ρ = 0.42; *P-*value = 0.085), and *Bifidobacterium* (ρ = 0.41; *P-*value = 0.088). Fructoselysine degradation correlated negatively with *Sutterella* (ρ = 0.52; *P-*value = 0.028). Carboxymethyllysine degradation was positively correlated with *Alistipes* (ρ = 0.49; *P-*value = 0.028) and *Akkermansia* (ρ = 0.47; *P-*value = 0.031). Detailed correlation plots of all statistically significant correlations are provided in Supporting Information Figure S11, while all genera correlated were additionally visualized in a heatmap in Supporting Information Figure S12.

## Discussion

**Figure 7:**
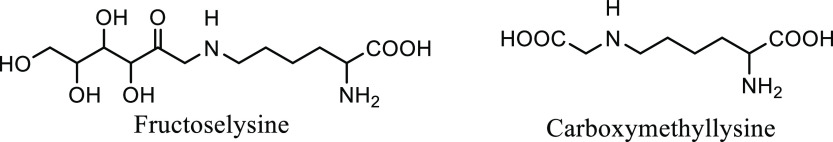
Chemical structures of fructoselysine and carboxymethyllysine presented in their free form.

In this study, we report interindividual and intraindividual differences in gut microbial degradation of the AGE carboxymethyllysine and its precursor fructoselysine (Figure 7). We show that fructoselysine is more readily degraded than carboxymethyllysine, and there appears to be no correlation between the degradation of the two.

Upon application of in vitro anaerobic incubations with individual human fecal slurries, pronounced interindividual differences in this microbial degradation capacity were found for both fructoselysine and carboxymethyllysine, ranging from no degradation at all to almost complete degradation of the substrates, added at saturating concentrations, under the employed experimental conditions. Interindividual differences in these microbial degradation capacities have been previously reported in the literature as well, although for a lower number of individuals, of which the experimentally obtained microbial degradation capacities were largely in line with our results.^22,23^ The substantial intraindividual differences for fructoselysine and carboxymethyllysine quantified in this study are, to the best of our knowledge, the first reported. Thus, this information on temporal variability within these degradation capacities, elucidated by analysis of fecal samples collected at different sampling times (3–16 weeks in between), could not be compared to studies in the literature.

Interindividual differences and the (in)ability to microbially degrade fructoselysine have been discussed previously,^26^ where the gene code yhfQ, coding for fructoselysine kinase involved in bacterial degradation of fructoselysine,^26^ was identified and shown to be present in the fecal metagenomes of only some individuals (∼10%).^26,31^ Via this pathway, fructoselysine can be phosphorylated into fructoselysine-6-phosphate^26^ which can be further metabolized by microbes and in some cases yield short-chain fatty acids (SCFAs) from it.^26^ However, the interindividual differences in the presence of this gene (yhfQ) only partially explain the quantified interindividual differences in fructoselysine degradation in the present study (since 95% of the tested fecal samples degraded the added fructoselysine at least to some extent). Another gene code coding for fructoselysine kinase has been identified as well (i.e., frlD^47,48^), which is involved in the microbial degradation of fructoselysine as well. Little has been reported about microbial degradation pathways of carboxymethyllysine. It is hypothesized that degradation pathways involve decarboxylase, oxidase, or 5-aminopentanamidase.^49^ However, this remains to be further investigated and confirmed.

Despite fructoselysine being a precursor for carboxymethyllysine, there was no correlation in the ability to microbially degrade the two substrates. Also, fructoselysine was degraded more efficiently than carboxymethyllysine as has been reported before,^22^ which, taken together, emphasizes that different metabolic pathways possibly present in different microbes are involved. In addition to this, the more efficient fructoselysine degradation might also be partly explained by a generally higher dietary exposure to fructoselysine (intake ± 7.1–14.3 mg/kg bw/day) compared to carboxymethyllysine (intake ± 0.3–1.1 mg/kg bw/day)^22,43^ and a potentially resulting microbial adaptation. This exposure-induced metabolic capacity is also proposed in a study where a small set of fecal metagenomes of breast fed and formula fed infants were analyzed for the presence of enzymes known to be involved in fructoselysine metabolism.^50^ Infants who consumed more formula, which, unlike breast milk, contains high levels of fructoselysine, had a higher expression of those degrading enzymes in their feces,^51^ indicating that pathways involved in fructoselysine metabolism can be induced by exposure. This corroborates with another study that reported that dietary exposure of mice to fructoselysine can influence the gut microbes themselves, which can again have possible effects on the bioremediation or degradation activity of certain members of the gut microbiota.^28^ The observed intraindividual differences in this study imply that possibly also in adults, fructoselysine degradation activities might be driven by exposure. However, this remains to be further investigated, for example, upon controlled dietary changes, and no conclusions on this matter can be derived from the present study since no detailed data on dietary consumption could be collected.

The observed inter- and intraindividual differences in degradation activities of the substrates are possibly partly due to differences in the abundances of specific bacterial species. A potential role was identified for the genera *Akkermansia*, *Megasphaera*, *Bifidobacterium,* and *Eubacterium_ruminantium_group* in fructoselysine degradation, while for carboxymethyllysine degradation, the genera *Alistipes* and *Akkermansia* might be involved based on our experimental results. In the literature, multiple bacteria have been reported to be involved in fructoselysine degradation (i.e., *Intestinimonas butyriciproducens*, *Bacillus subtilis,* and *E. coli*(26,47,48)) and carboxymethyllysine degradation (i.e., *E. coli*, *Oscillibacter*, and *Cloacibacillus evryenis*(25,49)). The variety of bacteria identified in the literature and the present study indicates that probably multiple bacteria in an ecosystem are responsible for the differences in microbial degradation activities instead of one specific bacteria. Based on the results of the present study alone, no causal relation between specific bacterial species and the degradation of fructoselysine and/or carboxymethyllysine can be made because of several reasons (e.g., low sample size, the limitations of 16S rRNA amplicon sequencing in bacteria identification) and was out of the scope of this study.

Some metabolites formed upon bacterial carboxymethyllysine degradation have been identified (i.e., 5-(carboxymethylamino)pentanoic acid, 2-amino-6-(formylmethylamino)hexanoic acid, carboxymethyl-cadaverine, and *N*-carboxymethyl-Δ^1^-piperideinium ion);^25,49^ however, this accounted for <10% of the concentrations of carboxymethyllysine actually being degraded. This reveals that probably other currently unknown metabolites are also formed upon carboxymethyllysine degradation. Possible SCFA formation has been hypothesized; however, in the present study, we could not experimentally confirm this (data not shown) partly due to high SCFA background levels in the fecal slurries of our experimental setup. For fructoselysine, the SCFA butyrate has been shown to be an important metabolite formed by *I. butyriciproducens,*(26) and butyrate production also correlated with fructoselysine degradation by human fecal slurries.^23^ Future studies on metabolite formation upon carboxymethyllysine and fructoselysine degradation are recommended to identify whether this degradation actually is a detoxification pathway as metabolites formed might be systemically available and can mediate effects of the gut microbiota on host health.^52^ In addition, it would be of interest to evaluate whether similar results on carboxymethyllysine and fructoselysine degradation can be obtained with real heat-processed foods and/or protein-bound glycation products as with the chemical standards as applied in the present study.

Inter- and intraindividual differences in fructoselysine and carboxymethyllysine gut microbial degradation can potentially affect internal exposure levels as not all individual tested fecal slurries were able to completely degrade the intake at the level of the EDI when extrapolating the in vitro obtained data to the in vivo situation. Quantification of interindividual differences in toxicokinetic data with the presented in vitro model might thus, depending on the research question, be a valuable contribution to human-based in vitro methodologies of modern toxicological risk assessment strategies as it can add to host metabolism. Altogether, the results of the present study show that the capacity for intestinal microbial degradation of these two compounds can be substantial, likely reducing internal exposure levels and thus the potential hazards related to dietary exposure of carboxymethyllysine and fructoselysine.

## Supporting Information Available

Fecal collection sampling times per individual; calculation of scaling of the in vitro data to the in vivo situation; optimization of experimental conditions for carboxymethyllysine; total bacterial cell load per gram fecal sample; amount of degraded carboxymethyllysine expressed per gram feces per hour; background concentrations in the human fecal slurries; amount of degraded substrate correlated with the total bacterial cell load per individual; amount of degraded fructoselysine expressed per gram feces per hour; linear regression between the amounts of degraded fructoselysine and carboxymethyllysine; PCoA of Bray–Curtis beta diversity of the microbial composition; absolute abundance of the genera in the fecal samples; relative abundance of microbial taxa in the fecal samples; correlation plots of the statistically significant correlated genera; and Spearman’s rank correlation analysis of bacterial genera with the amount of degraded fructoselysine and carboxymethyllysine quantified per gram feces (PDF)

## Abbreviations used

ACN: acetonitrile
AGE: advanced glycation end product
ASVs: amplicon sequence variants
CE: collision energy
CV: coefficient of variation
EDI: estimated daily intake
FDR: false discovery rate
LC-MS/MS: liquid-chromatography mass-spectrometry
MRM: multiple reaction monitoring
qPCR: quantitative polymerase chain reaction
rRNA: ribosomal RNA
SCFA: short chain fatty acid
ST: sampling time

## References

[ref1] Zhao D.; Sheng B.; Wu Y.; Li H.; Xu D.; Nian Y.; Mao S.; Li C.; Xu X.; Zhou G. Comparison of Free and Bound Advanced Glycation End Products in Food: A Review on the Possible Influence on Human Health. J. Agric. Food Chem. 2019, 67, 14007–14018. 10.1021/acs.jafc.9b05891. 31789029

[ref2] Poulsen M. W.; Hedegaard R. V.; Andersen J. M.; de Courten B.; Bügel S.; Nielsen J.; Skibsted L. H.; Dragsted L. O. Advanced Glycation Endproducts in Food and Their Effects on Health. Food Chem. Toxicol. 2013, 60, 10–37. 10.1016/J.FCT.2013.06.052. 23867544

[ref3] Maillard L. C. Action Des Acides Amines Sur Les Sucres; Formation Des Melanoidines Par Voie Methodique. C. R. Acad. Sci. 1912, 154, 66–68.

[ref4] Ahmed M. U.; Thorpe S. R.; Baynes J. W. Identification of N Epsilon-Carboxymethyllysine as a Degradation Product of Fructoselysine in Glycated Protein. J. Biol. Chem. 1986, 261, 4889 10.1016/s0021-9258(19)89188-3. 3082871

[ref5] Vistoli G.; De Maddis D.; Cipak A.; Zarkovic N.; Carini M.; Aldini G. Advanced Glycoxidation and Lipoxidation End Products (AGEs and ALEs): An Overview of Their Mechanisms of Formation. Free Radical Res. 2013, 47, 3–27. 10.3109/10715762.2013.815348. 23767955

[ref6] Glomb M. A.; Monnier V. M. Mechanism of Protein Modification by Glyoxal and Glycolaldehyde, Reactive Intermediates of the Maillard Reaction. J. Biol. Chem. 1995, 270, 10017–10026. 10.1074/jbc.270.17.10017. 7730303

[ref7] Vistoli G.; De Maddis D.; Cipak A.; Zarkovic N.; Carini M.; Aldini G. Advanced Glycoxidation and Lipoxidation End Products (AGEs and ALEs): An Overview of Their Mechanisms of Formation. Free Radical Res. 2013, 47, 3–27. 10.3109/10715762.2013.815348. 23767955

[ref8] Scheijen J.; Hanssen N. M. J.; van Greevenbroek M. M.; Van der Kallen C. J.; Feskens E. J. M.; Stehouwer C. D. A.; Schalkwijk C. G. Dietary Intake of Advanced Glycation Endproducts Is Associated with Higher Levels of Advanced Glycation Endproducts in Plasma and Urine: The CODAM Study. Clin. Nutr. 2018, 37, 919–925. 10.1016/j.clnu.2017.03.019. 29381139

[ref9] Uribarri J.; Cai W.; Peppa M.; Goodman S.; Ferrucci L.; Striker G.; Vlassara H. Circulating Glycotoxins and Dietary Advanced Glycation Endproducts: Two Links to Inflammatory Response, Oxidative Stress, and Aging. J. Gerontol., Ser. A 2007, 62, 427–433. 10.1093/gerona/62.4.427. PMC2645629 17452738

[ref10] Birlouez-Aragon I.; Saavedra G.; Tessier F. J.; Galinier A.; Ait-Ameur L.; Lacoste F.; Niamba C. N.; Alt N.; Somoza V.; Lecerf J. M. A Diet Based on High-Heat-Treated Foods Promotes Risk Factors for Diabetes Mellitus and Cardiovascular Diseases. Am. J. Clin. Nutr. 2010, 91, 1220–1226. 10.3945/ajcn.2009.28737. 20335546

[ref11] van Dongen K. C. W.; Linkens A. M. A.; Wetzels S. M. W.; Wouters K.; Vanmierlo T.; van de Waarenburg M. P. H.; Scheijen L. J. M.; Belzer W. M.; Schalkwijk C.; Schalkwijk C. G. Dietary Advanced Glycation Endproducts (AGEs) Increase Their Concentration in Plasma and Tissues, Result in Inflammation and Modulate Gut Microbial Composition in Mice; Evidence for Reversibility. Food Res. Int. 2021, 147, 110547 10.1016/J.FOODRES.2021.110547. 34399524

[ref12] Li M.; Zeng M.; He Z.; Zheng Z.; Qin F.; Tao G.; Zhang S.; Chen J. Effects of Long-Term Exposure to Free Nε-(Carboxymethyl)lysine on Rats Fed a High-Fat Diet. J. Agric. Food Chem. 2015, 63, 10995–11001. 10.1021/acs.jafc.5b05750. 26652688

[ref13] Tessier F. J.; Niquet-Léridon C.; Jacolot P.; Jouquand C.; Genin M.; Schmidt A.-M.; Grossin N.; Boulanger E. Quantitative assessment of organ distribution of dietary protein-bound13C-labeled Nε-carboxymethyllysine after a chronic oral exposure in mice. Mol. Nutr. Food Res. 2016, 60, 2446–2456. 10.1002/mnfr.201600140. 27393741

[ref14] Nowotny K.; Schröter D.; Schreiner M.; Grune T. Dietary Advanced Glycation End Products and Their Relevance for Human Health. Ageing Res. Rev. 2018, 47, 55–66. 10.1016/J.ARR.2018.06.005. 29969676

[ref15] Delgado-Andrade C.; Fogliano V. Dietary Advanced Glycosylation End-Products (DAGEs) and Melanoidins Formed through the Maillard Reaction: Physiological Consequences of Their Intake. Annu. Rev. Food Sci. Technol. 2018, 9, 271–291. 10.1146/annurev-food-030117-012441. 29350563

[ref16] Qu W.; Yuan X.; Zhao J.; Zhang Y.; Hu J.; Wang J.; Li J. Dietary Advanced Glycation End Products Modify Gut Microbial Composition and Partially Increase Colon Permeability in Rats. Mol. Nutr. Food Res. 2017, 61, 1700118 10.1002/mnfr.201700118. 28621836

[ref17] Seiquer I.; Rubio L. A.; Peinado M. J.; Delgado-Andrade C.; Navarro M. P. Maillard Reaction Products Modulate Gut Microbiota Composition in Adolescents. Mol. Nutr. Food Res. 2014, 58, 1552–1560. 10.1002/mnfr.201300847. 24867162

[ref18] Delgado-Andrade C.; Pastoriza de la Cueva S.; Peinado M. J.; Rufián-Henares J. Á.; Navarro M. P.; Rubio L. A. Modifications in Bacterial Groups and Short Chain Fatty Acid Production in the Gut of Healthy Adult Rats after Long-Term Consumption of Dietary Maillard Reaction Products. Food Res. Int. 2017, 100, 134–142. 10.1016/j.foodres.2017.06.067. 28873671

[ref19] Qu W.; Nie C.; Zhao J.; Ou X.; Zhang Y.; Yang S.; Bai X.; Wang Y.; Wang J.; Li J. Microbiome-Metabolomics Analysis of the Impacts of Long-Term Dietary Advanced-Glycation-End-Product Consumption on C57BL/6 Mouse Fecal Microbiota and Metabolites. J. Agric. Food Chem. 2018, 66, 8864–8875. 10.1021/acs.jafc.8b01466. 30037223

[ref20] Snelson M.; Tan S. M.; Clarke R. E.; de Pasquale C.; Thallas-Bonke V.; Nguyen T. V.; Penfold S. A.; Harcourt B. E.; Sourris K. C.; Lindblom R. S.; Ziemann M.; Steer D.; El-Osta A.; Davies M. J.; Donnellan L.; Deo P.; Kellow N. J.; Cooper M. E.; Woodruff T. M.; Mackay C. R.; Forbes J. M.; Coughlan M. T. Processed Foods Drive Intestinal Barrier Permeability and Microvascular Diseases. Sci. Adv. 2021, 7, 1–15. 10.1126/sciadv.abe4841. PMC8011970 33789895

[ref21] Mastrocola R.; Collotta D.; Gaudioso G.; Le Berre M.; Cento A. S.; Ferreira Alves G.; Chiazza F.; Verta R.; Bertocchi I.; Manig F.; Hellwig M.; Fava F.; Cifani C.; Aragno M.; Henle T.; Joshi L.; Tuohy K.; Collino M. Effects of Exogenous Dietary Advanced Glycation End Products on the Cross-Talk Mechanisms Linking Microbiota to Metabolic Inflammation. Nutrients 2020, 12, 2497 10.3390/nu12092497. PMC7551182 32824970

[ref22] Hellwig M.; Bunzel D.; Huch M.; Franz C. M. A. P.; Kulling S. E.; Henle T. Stability of Individual Maillard Reaction Products in the Presence of the Human Colonic Microbiota. J. Agric. Food Chem. 2015, 63, 6723–6730. 10.1021/acs.jafc.5b01391. 26186075

[ref23] van Dongen K. C. W.; van der Zande M.; Bruyneel B.; Vervoort J. J. M.; Rietjens I. M. C. M.; Belzer C.; Beekmann K. An in Vitro Model for Microbial Fructoselysine Degradation Shows Substantial Interindividual Differences in Metabolic Capacities of Human Fecal Slurries. Toxicol. In Vitro 2021, 72, 105078 10.1016/j.tiv.2021.105078. 33429044

[ref24] Bui T. P. N.; Troise A. D.; Fogliano V.; de Vos W. M. Anaerobic Degradation of N-ε-Carboxymethyllysine, a Major Glycation End-Product, by Human Intestinal Bacteria. J. Agric. Food Chem. 2019, 67, 6594–6602. 10.1021/acs.jafc.9b02208. 31091091 PMC6566499

[ref25] Hellwig M.; Auerbach C.; Müller N.; Samuel P.; Kammann S.; Beer F.; Gunzer F.; Henle T. Metabolization of the Advanced Glycation End Product N-ε-Carboxymethyllysine (CML) by Different Probiotic E. coli Strains. J. Agric. Food Chem. 2019, 67, 1963–1972. 10.1021/acs.jafc.8b06748. 30701968

[ref26] Bui T. P. N.; Ritari J.; Boeren S.; de Waard P.; Plugge C. M.; de Vos W. M. Production of Butyrate from Lysine and the Amadori Product Fructoselysine by a Human Gut Commensal. Nat. Commun. 2015, 6, 1–10. 10.1038/ncomms10062. PMC4697335 26620920

[ref27] Wiame E.; Delpierre G.; Collard F.; Van Schaftingen E. Identification of a Pathway for the Utilization of the Amadori Product Fructoselysine in *Escherichia Coli*. J. Biol. Chem. 2002, 277, 42523–42529. 10.1074/jbc.M200863200. 12147680

[ref28] Wolf A. R.; Wesener D. A.; Cheng J.; Houston-Ludlam A. N.; Beller Z. W.; Hibberd M. C.; Giannone R. J.; Peters S. L.; Hettich R. L.; Leyn S. A.; Rodionov D. A.; Osterman A. L.; Gordon J. I. Bioremediation of a Common Product of Food Processing by a Human Gut Bacterium. Cell Host Microbe 2019, 26, 463–477. 10.1016/j.chom.2019.09.001. 31585844 PMC6801109

[ref29] van Dongen K. C. W.; Kappetein L.; Miro Estruch I.; Belzer C.; Beekmann K.; Rietjens I. M. C. M. Differences in Kinetics and Dynamics of Endogenous versus Exogenous Advanced Glycation End Products (AGEs) and Their Precursors. Food Chem. Toxicol. 2022, 164, 112987 10.1016/J.FCT.2022.112987. 35398182

[ref30] Rothschild D.; Weissbrod O.; Barkan E.; Kurilshikov A.; Korem T.; Zeevi D.; Costea P. I.; Godneva A.; Kalka I. N.; Bar N.; Shilo S.; Lador D.; Vila A. V.; Zmora N.; Pevsner-Fischer M.; Israeli D.; Kosower N.; Malka G.; Wolf B. C.; Avnit-Sagi T.; Lotan-Pompan M.; Weinberger A.; Halpern Z.; Carmi S.; Fu J.; Wijmenga C.; Zhernakova A.; Elinav E.; Segal E. Environment Dominates over Host Genetics in Shaping Human Gut Microbiota. Nat 2018, 555, 210–215. 10.1038/nature25973. 29489753

[ref31] Huttenhower C.; Gevers D.; Knight R.; et al. Structure, Function and Diversity of the Healthy Human Microbiome. Nature 2012, 486, 207–214. 10.1038/nature11234. 22699609 PMC3564958

[ref32] Garud N. R.; Good B. H.; Hallatschek O.; Pollard K. S. Evolutionary Dynamics of Bacteria in the Gut Microbiome within and across Hosts. PLoS Biol. 2019, 17, e3000102 10.1371/JOURNAL.PBIO.3000102. 30673701 PMC6361464

[ref33] Wilms E.; An R.; Smolinska A.; Stevens Y.; Weseler A. R.; Elizalde M.; Drittij M. J.; Ioannou A.; van Schooten F. J.; Smidt H.; Masclee A. A. M.; Zoetendal E. G.; Jonkers D. M. A. E. Galacto-Oligosaccharides Supplementation in Prefrail Older and Healthy Adults Increased Faecal Bifidobacteria, but Did Not Impact Immune Function and Oxidative Stress. Clin. Nutr. 2021, 40, 3019–3031. 10.1016/j.clnu.2020.12.034. 33509667

[ref34] Větrovský T.; Baldrian P. The Variability of the 16S RRNA Gene in Bacterial Genomes and Its Consequences for Bacterial Community Analyses. PLoS One 2013, 8, e57923 10.1371/JOURNAL.PONE.0057923. 23460914 PMC3583900

[ref35] Poncheewin W.; Hermes G. D. A.; van Dam J. C. J.; Koehorst J. J.; Smidt H.; Schaap P. J. NG-Tax 2.0: A Semantic Framework for High-Throughput Amplicon Analysis. Front. Genet. 2020, 10, 10 10.3389/fgene.2019.01366. PMC6989550 32117417

[ref36] Quast C.; Pruesse E.; Yilmaz P.; Gerken J.; Schweer T.; Yarza P.; Peplies J.; Glöckner F. O. The SILVA Ribosomal RNA Gene Database Project: Improved Data Processing and Web-Based Tools. Nucleic Acids Res. 2013, 41, D590–D596. 10.1093/nar/gks1219. 23193283 PMC3531112

[ref37] McMurdie P. J.; Holmes S. Phyloseq: An R Package for Reproducible Interactive Analysis and Graphics of Microbiome Census Data. PLoS One 2013, 8, e61217 10.1371/journal.pone.0061217. 23630581 PMC3632530

[ref38] Lahti L.; Shetty S. A.; Tools for microbiome analysis in R. http://microbiome.github.com/microbiome (accessed on Jun 15, 2020).

[ref39] Paradis E.; Claude J.; Strimmer K. APE: Analyses of Phylogenetics and Evolution in R Language. Bioinformatics 2004, 20, 289–290. 10.1093/BIOINFORMATICS/BTG412. 14734327

[ref40] Jian C.; Luukkonen P.; Yki-Järvinen H.; Salonen A.; Korpela K. Quantitative PCR Provides a Simple and Accessible Method for Quantitative Microbiota Profiling. PLoS One 2020, 15, e0227285 10.1371/JOURNAL.PONE.0227285. 31940382 PMC6961887

[ref41] Wilson C. G. Gastrointestinal Transit and Drug Absorption. In Oral Drug Absorption Prediction and Assessment; Dressman J. B., Lennernas H., Eds.; 2000; pp 1–17.

[ref42] Rose C.; Parker A.; Jefferson B.; Cartmell E. The Characterization of Feces and Urine: A Review of the Literature to Inform Advanced Treatment Technology. Crit. Rev. Environ. Sci. Technol. 2015, 45, 1827–1879. 10.1080/10643389.2014.1000761. 26246784 PMC4500995

[ref43] Henle T. AGEs in Foods: Do They Play a Role in Uremia?. Kidney Int. 2003, 63, S145 10.1046/j.1523-1755.63.s84.16.x. 12694332

[ref44] Falony G.; Joossens M.; Vieira-Silva S.; Wang J.; Darzi Y.; Faust K.; Kurilshikov A.; Bonder M. J.; Valles-Colomer M.; Vandeputte D.; Tito R. Y.; Chaffron S.; Rymenans L.; Verspecht C.; De Sutter L.; Lima-Mendez G.; D’hoe K.; Jonckheere K.; Homola D.; Garcia R.; Tigchelaar E. F.; Eeckhaudt L.; Fu J.; Henckaerts L.; Zhernakova A.; Wijmenga C.; Raes J. Population-Level Analysis of Gut Microbiome Variation. Science 2016, 352, 560–564. 10.1126/SCIENCE.AAD3503/SUPPL_FILE/TABLE_S9.XLSX. 27126039

[ref45] Schlomann B. H.; Parthasarathy R. Timescales of Gut Microbiome Dynamics. Curr. Opin. Microbiol. 2019, 50, 56–63. 10.1016/J.MIB.2019.09.011. 31689582 PMC6899164

[ref46] Mehta R. S.; Abu-Ali G. S.; Drew D. A.; Lloyd-Price J.; Subramanian A.; Lochhead P.; Joshi A. D.; Ivey K. L.; Khalili H.; Brown G. T.; DuLong C.; Song M.; Nguyen L. H.; Mallick H.; Rimm E. B.; Izard J.; Huttenhower C.; Chan A. T. Stability of the Human Faecal Microbiome in a Cohort of Adult Men. Nat. Microbiol. 2018, 3, 347–355. 10.1038/s41564-017-0096-0. 29335554 PMC6016839

[ref47] Wiame E.; Delpierre G.; Collard F.; Van Schaftingen E. Identification of a Pathway for the Utilization of the Amadori Product Fructoselysine in Escherichia Coli. J. Biol. Chem. 2002, 277, 42523–42529. 10.1074/jbc.M200863200. 12147680

[ref48] Wiame E.; Duquenne A.; Delpierre G.; Van Schaftingen E. Identification of enzymes acting on α-glycated amino acids in Bacillus subtilis. FEBS Lett. 2004, 577, 469–472. 10.1016/j.febslet.2004.10.049. 15556630

[ref49] Bui T. P. N.; Troise A. D.; Fogliano V.; de Vos W. M. Anaerobic Degradation of N-ε-Carboxymethyllysine, a Major Glycation End-Product, by Human Intestinal Bacteria. J. Agric. Food Chem. 2019, 67, 6594–6602. 10.1021/acs.jafc.9b02208. 31091091 PMC6566499

[ref50] Bui T. P. N.; Troise A. D.; Nijsse B.; Roviello G. N.; Fogliano V.; de Vos W. M. Intestinimonas-like bacteria are important butyrate producers that utilize Nε-fructosyllysine and lysine in formula-fed infants and adults. J. Funct. Foods 2020, 70, 103974 10.1016/j.jff.2020.103974.

[ref51] Sillner N.; Walker A.; Hemmler D.; Bazanella M.; Heinzmann S. S.; Haller D.; Schmitt-Kopplin P. Milk-Derived Amadori Products in Feces of Formula-Fed Infants. J. Agric. Food Chem. 2019, 67, 8061–8069. 10.1021/acs.jafc.9b01889. 31264412

[ref52] Chen L.; Wang D.; Garmaeva S.; Kurilshikov A.; Vich Vila A.; Gacesa R.; Sinha T.; Segal E.; Weersma R. K.; Wijmenga C.; Zhernakova A.; Fu J. The Long-Term Genetic Stability and Individual Specificity of the Human Gut Microbiome. Cell 2021, 184, 2302–2315. 10.1016/J.CELL.2021.03.024. 33838112

[ref53] Brown R. P.; Delp M. D.; Lindstedt S. L.; Rhomberg L. R.; Beliles R. P. Physiological Parameter Values for Physiologically Based Pharmacokinetic Models. Toxicol. Ind. Health 1997, 13, 407–484. 10.1177/074823379701300401. 9249929

