# Supplementary material for: Inter- and Intraindividual Differences in the Capacity of the Human Intestinal Microbiome in Fecal Slurries to Metabolize Fructoselysine and Carboxymethyllysine
Source: J Agric Food Chem. 2022 Sep 7;70(37):11759–68. doi: 10.1021/acs.jafc.2c05756 (PMC9501902; doi:10.1021/acs.jafc.2c05756)
Supplement: Supplementary file 1 — jf2c05756_si_001.pdf [file jf2c05756_si_001.pdf]

Supporting information

Inter- and intraindividual differences in the capacity of the human intestinal microbiome in fecal slurries to metabolize fructoselysine and carboxymethyllysine

Katja C.W. van Dongen[1*], Clara Belzer[2], Wouter Bakker[1], Ivonne M.C.M. Rietjens[1], Karsten Beekmann[3]

1. Division of Toxicology, Wageningen University and Research, P.O. Box 8000, 6700 EA, Wageningen, the Netherlands
2. Laboratory of Microbiology, Wageningen University and Research, P.O. Box 8033, 6700 EH, Wageningen, the Netherlands
3. Wageningen Food Safety Research (WFSR), part of Wageningen University and Research, P.O. Box 230, 6700 AE, Wageningen, the Netherlands

Supporting information

Supplementary Tables

Table S1 *Fecal collection sampling times per individual. Fecal samples were collected at three sampling times (i.e. ST1, ST2 and ST3) of which ST1 is referred to as the first sampling time and indicated as week 0. The Δt shows the number of weeks the sample was donated compared to ST1.*

Individual	ST2 (Δt compared to ST1 in weeks)	ST3 (Δt compared to ST1 in weeks)
1	9	12
2	3	6
3	3	16
4	3	6
5	6	9
6	6	9
7	3	6
8	3	6
9	6	9
10	3	6
11	3	9
12	3	9
13	3	6

***Table S2** Calculation of scaling the in vitro determined degradation parameters to the in vivo situation*

	Daily intake (mg/kg bw)	Daily intake (µmol)[a]	In vitro degradation capacities per hour[b,c]	In vitro degradation capacities per 24 hours[b,d]	In vivo degradation capacity (µmol)[e]	% Of transit time for complete degradation of the daily intake	Number of incomplete degradations out of 46 individuals[h]	Number of incomplete degradations out of 20 individuals[i]
Fructose-lysine[f]	7.1-14.3	1612.1-3246.8	1.64	39.44	5048.13	31.9-64.3	8-20	4-10
Carboxy-methyl-lysine[g]	0.3-1.1	102.8-376.9	0.07	1.69	216.01	47.6-174.5	18-39	11-19

[a] Assuming an average body weight for adults of 70 kg[53].
[b] Expressed as µmol/g feces/h.
[c] Degradation capacities were experimentally obtained in the present study and represent the average of all tested fecal samples excluding the outlier Individual 1 ST1.
[d] Transformed to total transit time in the colon which equals 24 hours[41].
[e] A total fecal mass of 128 grams per 24 hours was assumed[42].
[f] Molecular weight is 308.3 g/mol.
[g] Molecular weight is 204.2 g/mol.
[h]Number of individual collected fecal samples tested (n=46) with degradation activities[b] too low to completely degrade the daily intake[a]
[i]Number of individuals who donated a fecal sample at least once (n=20) with one or more sampling times (i.e. ST1, ST2 and/or ST3) with degradation activities[b] too low to completely degrade the daily intake[a]

Supplementary Figures

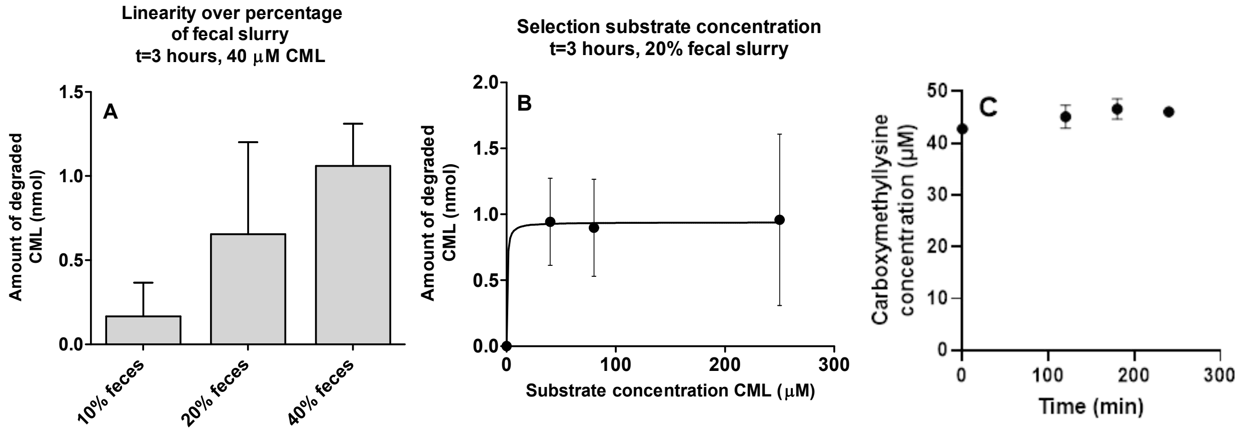

Figure S1 *Optimization of experimental conditions using pooled human fecal slurries. Linearity of carboxymethyllysine (CML) degradation over percentage of fecal concentration at 3 hours of anaerobic incubation with 40 µM CML (A) and selection of a saturated substrate concentration of CML (µM) at 3 hours incubation using 20% pooled human fecal concentration. Error bars show the standard deviation of each data point. (C) Shows the stability of carboxymethyllysine in PBS over time in anaerobic incubations without addition of human fecal slurry.*

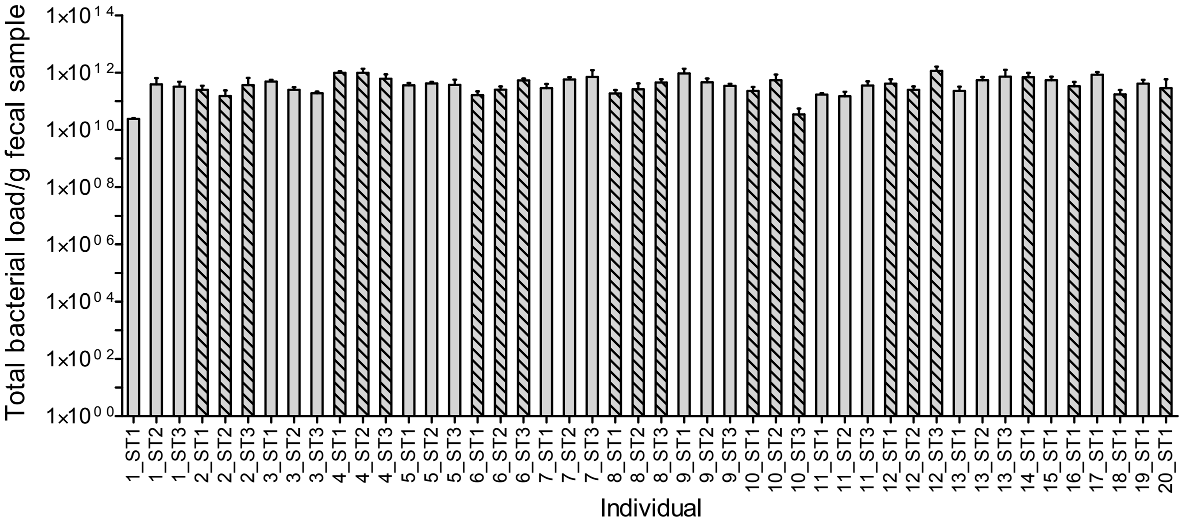

Figure S2 *Total bacterial cell load per gram fecal sample, as collected per individual (indicated by initial numbers on x-axis labels) at different sampling times (i.e. ST1, ST2 and ST3). Total bacterial cell load was determined by qPCR, and data represent the average ± SD of three technical replicates.*

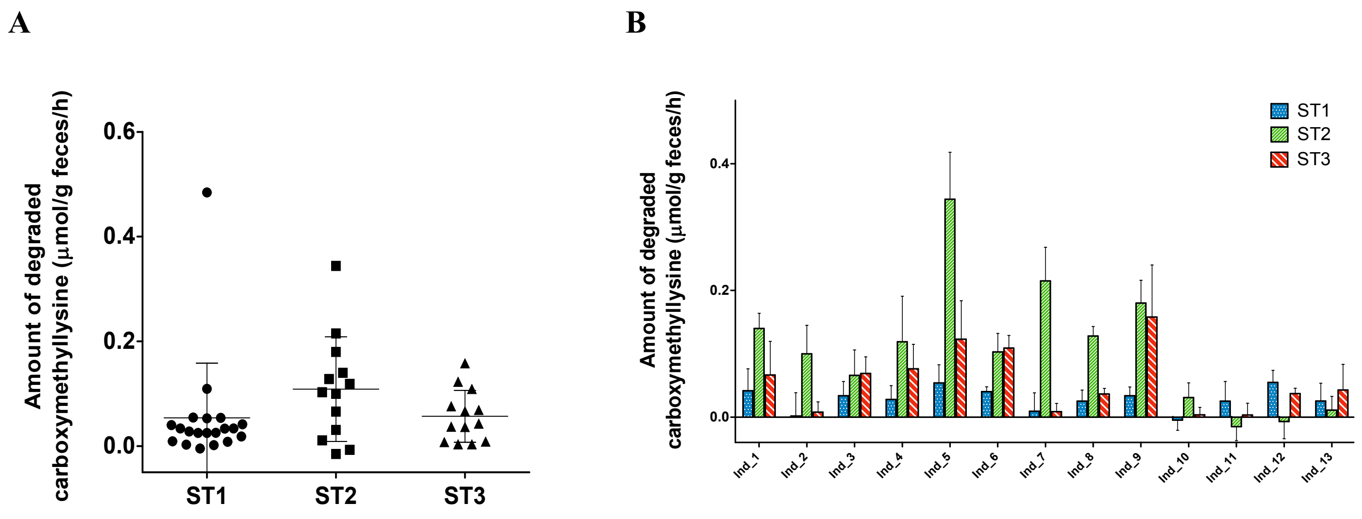

Figure S3 *Amount of carboxymethyllysine degraded after anaerobic incubation of individual human fecal samples (0.05 g/ml final concentration) with 80 µM carboxymethyllysine, expressed per hour. ST1, ST2 and ST3 indicate different sampling times, ST1 includes 20 individual donated fecal samples, ST2 and ST3 each 13 individually donated fecal samples. Scatter plots (A) show general spread in the populations with the center bars of the scatter dots indicating mean values while whiskers indicate the SD. Bar plot (B) shows intraindividual differences in carboxymethyllysine degradation of 13 individual donors sampled at the three sampling times. All data points represent average values of three independent experiments.*

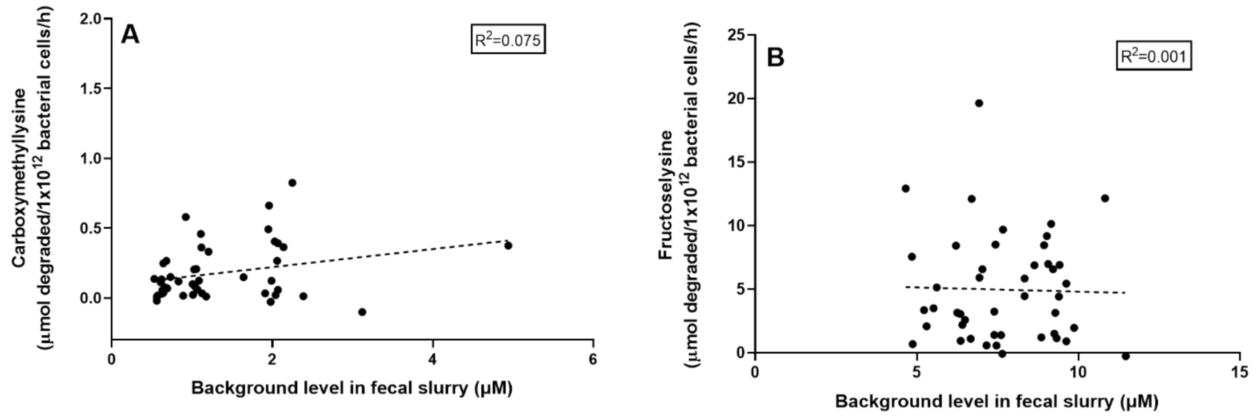

Figure S4 *Background concentrations of (A) carboxymethyllysine and (B) fructoselysine in the human fecal slurries applied in the anaerobic incubations, which were correlated (linear regression) with individuals' degradation activity. Each data point represents one individual at one sampling time.*

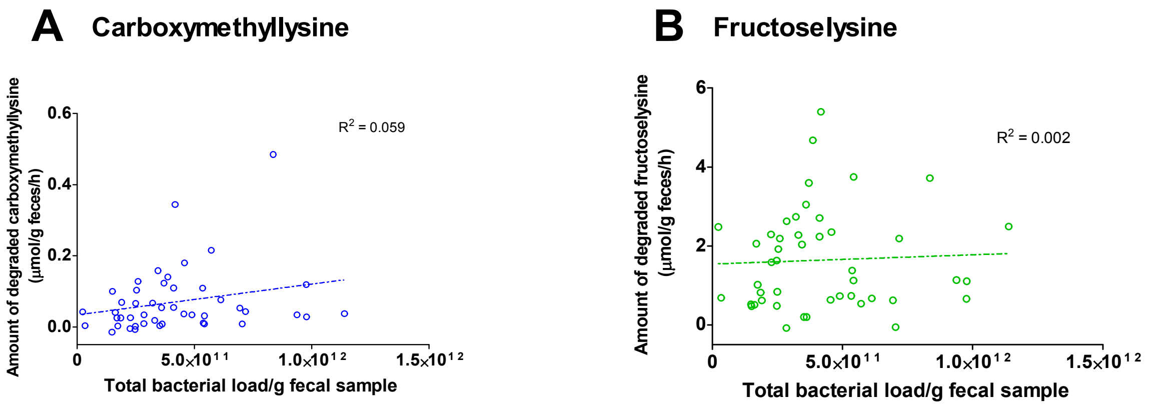

Figure S5 *Amount of degraded carboxymethyllysine (A) and fructoselysine (B) per hour of anaerobic incubation per gram feces correlated to the total bacterial load per gram fecal sample of each individual tested. Data points of the degradation represent an average value of three independent experiments.*

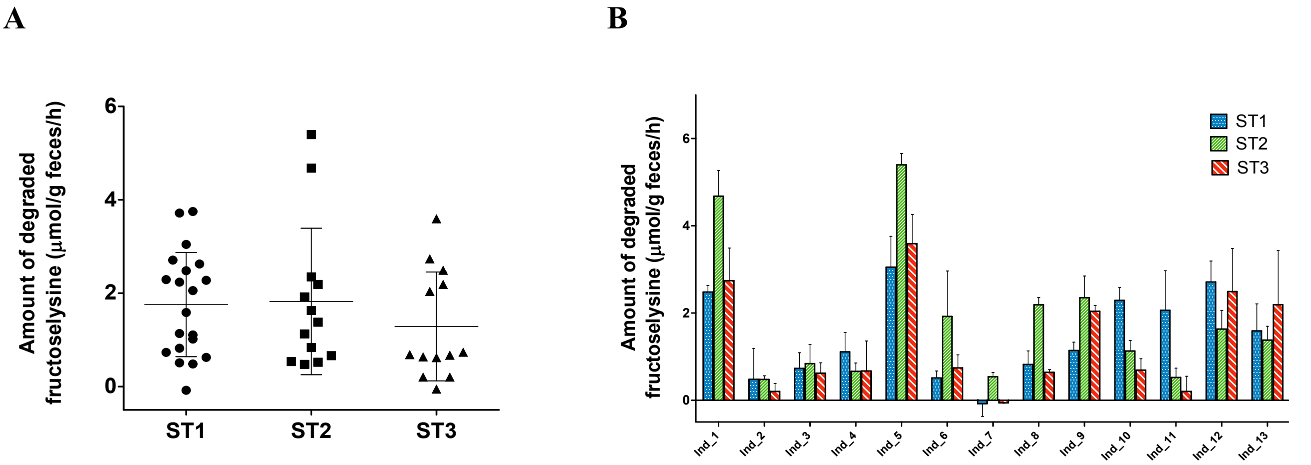

Figure S6 *Amount of fructoselysine degraded after anaerobic incubation of individual human fecal samples (0.0125 g/mL final concentration) with 125 µM fructoselysine per hour. ST1, ST2 and ST3 indicate different sampling times; ST1 includes 20 individual donated fecal samples, ST2 and ST3 each 13 individually donated fecal samples. Scatter plots (A) show general spread in the populations with the center bars of the scatter dots indicating mean values while whiskers indicate the SD. Bar plot (B) shows intraindividual differences in fructoselysine degradation of 13 individual donors sampled at the three sampling times. All data points represent average values of three independent experiments.*

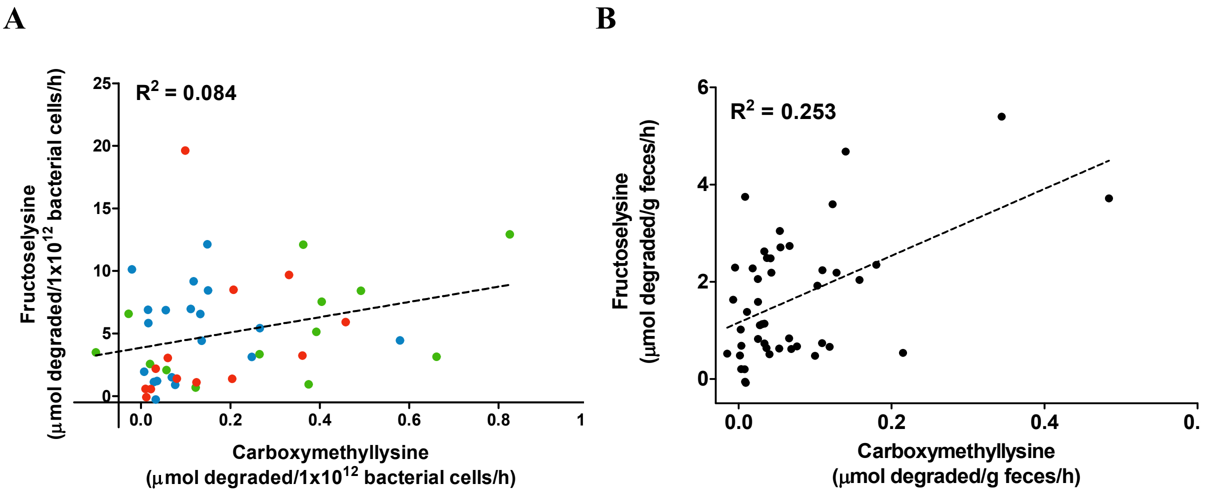

Figure S7 *Linear regression between the amount of fructoselysine and carboxymethyllysine being degraded by anaerobic incubations with individual human fecal slurries, (A) presented as µmol degraded/1x10^{12} bacterial cells/h and (B) presented as µmol degraded/g feces per hour. A total of 46 samples were included consisting of 20 individuals (sampling time ST1) whereof 13 individuals donated additionally twice more a fecal sample (sampling times ST2 and ST3). In (A) blue circles refer to ST1; green circles to ST2; red circles to ST3. Individual 1, ST1 was excluded from the analysis as it was assessed as an outlier.*

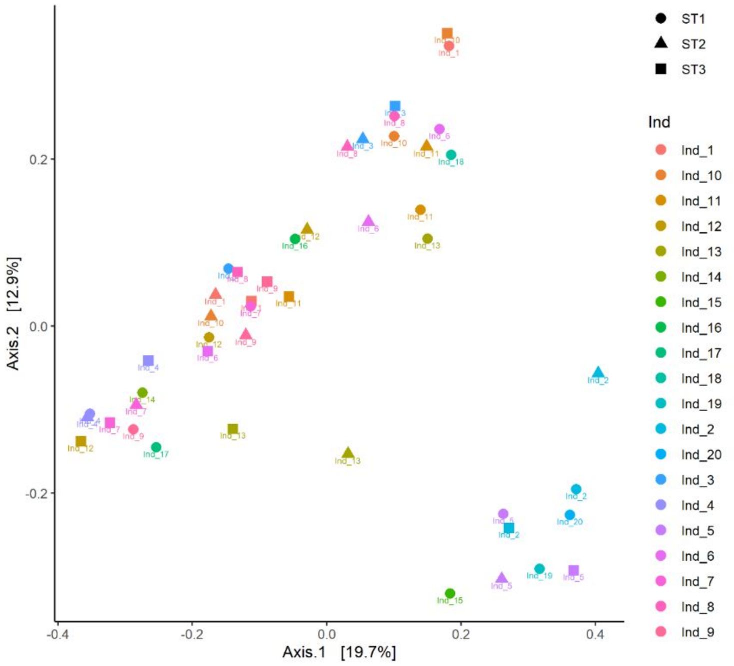

Figure S8 *PCoA plot of Bray-Curtis beta diversity dissimilarities of 20 individual (Ind) collected human fecal samples (i.e. ST1), of which 13 individuals donated at two additional sampling times (i.e. ST2, ST3). Each data point represents one fecal sample, labelled and colored with the individual number and the different symbols refer to the three sampling times.*

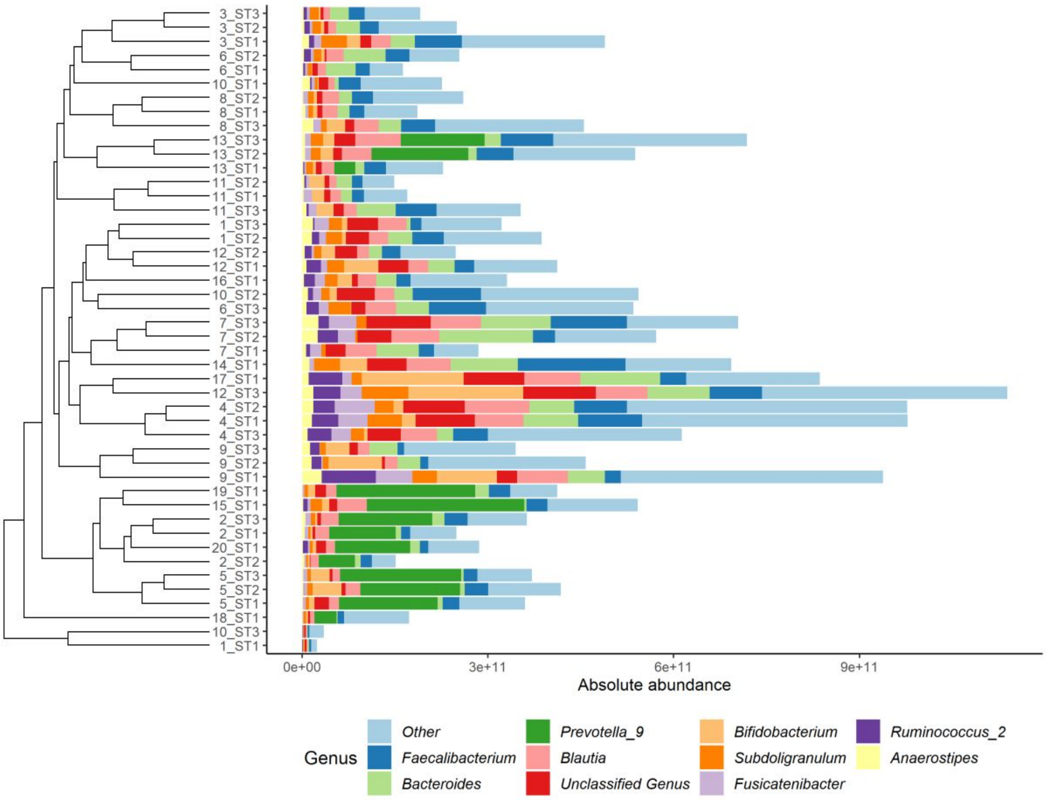

Figure S9 *Absolute abundance of microbial taxa, assessed with 16S rRNA sequencing and qPCR, present in the individual fecal samples (y-axis labels consist of subject number and sampling time). The top 10 taxa present at genus level are shown, sorted based on hierarchical clustering of Bray-Curtis dissimilarities using the average linkage approach with all taxa included.*

Supporting information

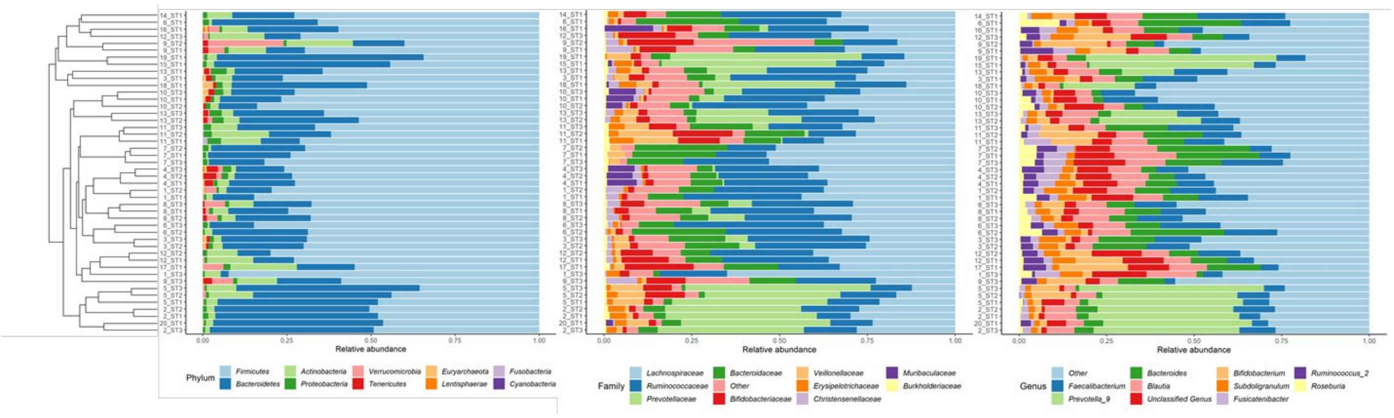

Figure S10 *Relative abundance of microbial taxa, assessed with 16S rRNA sequencing, present in the individual fecal samples (y-axis labels consist of subject number and sampling time). The top 10 taxa present at phylum, family and genus level are shown, sorted based on hierarchical clustering of Bray-Curtis dissimilarities using the average linkage approach with all taxa included.*

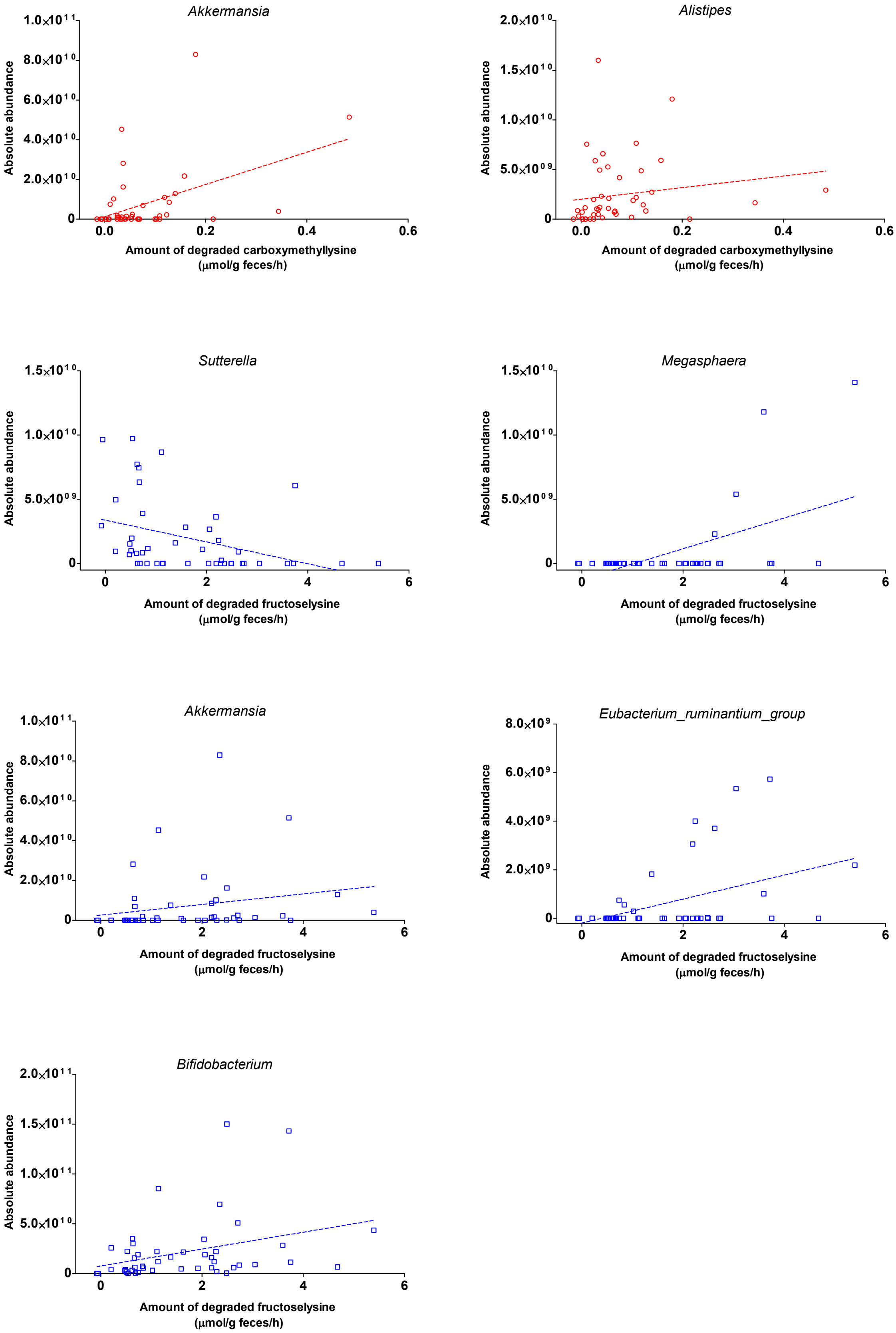

Figure S11 *Detailed correlation plots of the statistically significant correlated genera with carboxymethyllysine (red) and fructoselysine (blue) degradation.*

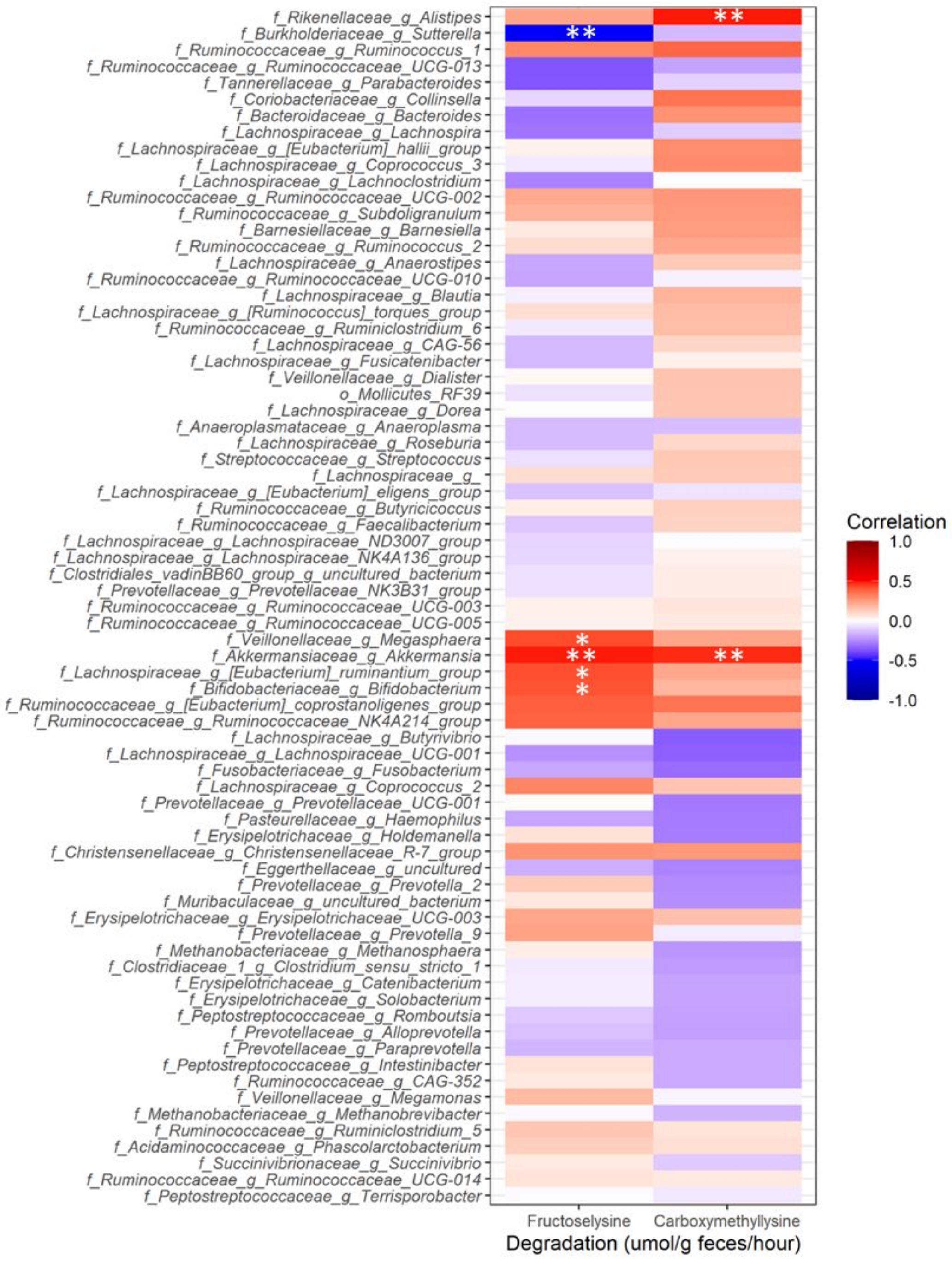

Figure S12 *Spearman's rank correlation analysis of bacterial genera with the amount of degraded fructoselysine and carboxymethyllysine as quantified per gram feces used in the incubation system, expressed per hour. Bacterial genera present with a relative abundance >1% in one of the fecal samples were included and were transformed into absolute abundance (using quantified total bacterial cell load by qPCR). Statistically significant correlations after correction for multiple testing (FDR) were indicated as follows: ** p-value < 0.05; * p-value <0.1.*
